# Conserved regions upstream of *BRC1B* regulate bud dormancy in tomato

**DOI:** 10.3389/fpls.2025.1702139

**Published:** 2025-11-20

**Authors:** Gül Hatinoğlu, Froukje van der Wal, Gerco C. Angenent, Ruud A. de Maagd, Richard G.H. Immink

**Affiliations:** 1 Laboratory of Molecular Biology, Wageningen University & Research, Wageningen, Netherlands; 2 Bioscience, Wageningen Plant Research, Wageningen University & Research, Wageningen, Netherlands; 3 Laboratory of Cell & Developmental Biology, Wageningen University & Research, Wageningen, Netherlands

**Keywords:** *BRANCHED1*, shoot branching, axillary bud, cis-regulation, quantitative trait, CRISPR/Cas9

## Abstract

Plant architecture is majorly influenced by shoot branching through the development of axillary meristems in the leaf axils. These meristems develop into axillary buds, which are kept dormant until endogenous or exogenous cues allow their activation. The key TCP class II transcription factor (TF) BRANCHED1 (BRC1) regulates the early stages of bud outgrowth. *BRC1* expression is highly specific to axillary buds, where it inhibits outgrowth by integrating hormonal, nutrient, and environmental signals. While the function of *BRC1/TB1* genes is highly conserved in numerous species, the *cis*- and *trans*-regulation of *BRC1* expression remains poorly understood. In this study, we explored how modifications in the *SlBRC1B* promoter affect bud outgrowth in tomato. We identified four highly conserved regions (*CR1 to CR4*) in the sequence upstream of the *SlBRC1B* translation start site by performing a phylogenetic footprinting. A collection of promoter mutants was generated by separately targeting each *CR* using CRISPR/Cas9. These *CR* mutants were employed for a detailed bud outgrowth characterization to investigate the effect of mutations on SlBRC1B-mediated bud dormancy. Most *CR* mutants consistently showed decreased bud outgrowth, suggesting that *SlBRC1B* is under tight control of various transcriptional repressors. Screening *CR4* with an axillary bud-specific cDNA library in a Yeast one-hybrid assay identified MYB, GRF, NAC, MADS, and zinc finger TF family members. Based on our findings, we concluded that the identified *CRs* play a crucial role in regulating *SlBRC1B* expression, and that they could be strategically targeted to achieve a desired level of shoot branching.

## 1 Introduction

Shoot branching plays a crucial role in plant architecture, in which the branching pattern relies on the development and growth of axillary shoots. Axillary shoot development starts with the formation of a meristem followed by the development of leaf primordia, comprising the axillary bud. These buds remain dormant until favorable endogenous or exogenous conditions trigger their outgrowth (Bennett and Leyser, 2006; McSteen and Leyser, 2005; Schmitz and Theres, 2005). This dormancy is maintained by apical dominance and removing the growing apex (decapitation) disrupts it, allowing bud outgrowth (Cline, 1997; Dun et al., 2006; Srivastava, 2002; Thimann and Skoog, 1933). The transition from dormant to active bud is a developmental process that is conserved across different plant species, and it is achieved by various endogenous and environmental signals converging on a *BRANCHED1 (BRC1)*-dependent pathway (Aguilar-Martínez et al., 2007; Choi et al., 2012; Finlayson, 2007; González-Grandío et al., 2013; Studer et al., 2011). Thus, BRC1 serves as a pivotal factor in integrating various signals for bud outgrowth.

In many plants, the expression of the class II TEOSINTE BRANCHED 1/CYCLOIDEA/PCF1 (TCP) transcription factor (TF) *BRC1* is highly specific to the axillary buds where it suppresses their outgrowth (Rameau et al., 2015; Wang et al., 2019). The *BRC1* ortholog *TEOSINTE BRANCHED 1 (TB1)* in maize was first discovered due to elevated *TB1* expression compared to its wild relative teosinte. This increased expression reduced shoot branching from the basal nodes and shifted the development of female flowers to male in reproductive meristems (Clark et al., 2006; Doebley et al., 1995; Stitzer and Ross-Ibarra, 2018). Knocking out *TB1* in maize (*ZmTB1*) and rice (*OsTB1*), on the other hand, resulted in increased branching (Choi et al., 2012; Studer et al., 2011). Similarly, in dicots, knockout mutants of *BRC1*, such as in Arabidopsis showed increased branching from the axils of rosette leaves (Aguilar-Martínez et al., 2007). In tomato, the knockdown of *SlBRC1B*, one of two BRC1 orthologs, also increased the axillary shoot length at the basal nodes (Martín-Trillo et al., 2011) as well as the total number of developing axillary shoots (Dong et al., 2023). Consistent with these findings, basal buds exhibited the most prominent *SlBRC1B* expression (Martín-Trillo et al., 2011). These findings underscore the function of BRC1 as a repressor of bud outgrowth.

Transcriptional regulation is mediated by the TFs that directly bind to regulatory sequences known as *cis*-regulatory elements (CREs) within a gene locus. These CREs play a crucial role in activating or repressing transcription and are suggested to be conserved throughout evolution (Lieberman-Lazarovich et al., 2019; Maeso et al., 2013; Nitta et al., 2015). Identifying such CREs in key developmental regulators, including *WUSCHEL (WUS)*, *CLAVATA3 (CLV3)*, and BRC1/*TB1*, is challenging, but has become a powerful strategy to pinpoint functional regulatory sequences underlying developmental and domestication traits across different plant species (Meyer and Purugganan, 2013; Olsen and Wendel, 2013; Swinnen et al., 2016). Although numerous endogenous and environmental signals affect *BRC1/TB1* expression (Barbier et al., 2019; Wang et al., 2019), only a few CREs upstream of *BRC1/TB1* have been identified, e.g., in rice and Arabidopsis (Lu et al., 2013; Xie et al., 2020). In tomato, only the binding sites for BRASSINAZOLE-RESISTANT 1 (BZR1) and ELONGATED HYPOCOTYL 5 (HY5) have been identified upstream of *SlBRC1B* and were shown to be bound by the respective transcription factors (Dong et al., 2023; Xia et al., 2021). Despite these findings providing insights into the regulatory sequences upstream of *BRC1*/*TB1* genes, the precise mechanism by which endogenous and exogenous signals impact the *BRC1* promoter activity remains unknown. Therefore, the specific *in vivo* function of CREs and undiscovered regulatory sequences requires further study to understand the complex regulatory network of BRC1-dependent bud outgrowth.

In this study, we investigated the potential tomato *BRC1B* regulatory regions (promoter and terminator) to shed light on *SlBRC1B* regulation in axillary buds. Since *BRC1* genes across different species show very conserved expression patterns, we hypothesized that this could indicate a conserved *cis-trans* regulatory landscape. Using comparative genomics with orthologous sequences, we identified four highly conserved regions (*CRs*) upstream of *SlBRC1B*. Subsequently, we modified these *CR*s by CRISPR/Cas9-mutagenesis, resulting in significant changes in bud outgrowth. Yeast one-hybrid screens with individual *CR*s resulted in the identification of GRF, MYB, NAC, zinc finger, and MADS TFs as potential regulators of its expression. We concluded that the *CR*s contain CREs that are critical for precise *SlBRC1B* regulation.

## 2 Materials and methods

### 2.1 Bioinformatics

*SlBRC1B* homologs were initially identified by screening their coding sequences (CDS) using BLASTp with default settings (BLOSUM62, first 100 hits) against the NCBI database (https://blast.ncbi.nlm.nih.gov/Blast.cgi). In Solanaceae, both closely related *BRC1A*- and *BRC1B*-subclades were identified and *BRC1* homologous sequences from species such as Arabidopsis and cucumber were included for the conservation analysis. Five kb upstream of *BRC1A*-, *BRC1B*- subclade members and cucumber genes, and two kb upstream of both Arabidopsis copies (*AtBRC1*, *AtBRC2*) were retrieved from NCBI database (https://www.ncbi.nlm.nih.gov/). These sequences were investigated using mVISTA with the LAGAN algorithm employing either a 50- or 100-bp window (Brudno et al., 2003; Frazer et al., 2004). These settings are referred to as readjusted or default and are specified in the figure legends. Potential upstream open reading frames were predicted using the Expasy Translate tool (https://web.expasy.org/translate/). For motif analysis, five kb upstream regions were examined with MEME for any number of repetitions with a maximum of ten motif sites (Bailey et al., 2015). To identify consensus binding sites, the MEME-identified motif sites were screened against the Arabidopsis DAP-seq database using the TOMTOM algorithm (Bailey et al., 2009).

### 2.2 Plant material and growth conditions

Tomato (*Solanum lycopersicum* cv. Moneyberg) seeds were sown on filter paper in the dark at 21°C for five days for germination. Seedlings were transferred to rockwool and grown in growth chamber conditions (21°C; 16 hours/8 hours light/dark) for a short period. T_0_, T_1_ and T_2_ mutant plants were moved to a greenhouse compartment (ambient temperature > 20°C; 16 hours/8 hours light/dark supplemented with sodium light) for seed production.

For phenotyping and gene expression experiments, some modifications were applied. Germinated seedlings were transferred to large rockwool blocks (15 cm x 15 cm) seven days after sowing (7 DAS) and directly placed on tables in the greenhouse. Plants were randomized and spaced 15 cm or 10 cm apart for phenotyping and bud harvesting, respectively, to avoid shade responses.

### 2.3 CRISPR/Cas9 mutagenesis

For each conserved region (*CR*), four specific sgRNAs were selected by comparing sgRNA on- and off-target efficiency scores with online available tools CRISPOR (Concordet and Haeussler, 2018), CRISPR-p (Lei et al., 2014) and CHOPCHOP (Labun et al., 2019). The most (predicted) effective four sgRNAs with PAM sequence NGG and U6–26 promoter-compatibility were selected based on Doench et al. (2016) and Moreno-Mateos et al. (2015) algorithms. Selected sgRNAs were cloned into the level1 (L1) CRISPR-pink Golden Gate-compatible vectors previously constructed by Slaman et al. (2023). L1 vectors containing the human codon-optimized SpCas9, marker genes such as NPTII and GFP together with four sgRNAs were combined into the final L2 vector. The final L2 construct was transformed to *Agrobacterium tumefaciens* strain C58C1 for plant transformation. The primers used for cloning can be found in **Supplementary Table S4**.

### 2.4 Plant transformation

The tomato plant transformation protocol by van Roekel et al. (1993) was followed with modifications. Explants were incubated with *Agrobacterium* suspensions for 20 minutes in the petri dish and gently swirled. Next, the suspension was removed, and the explants were transferred to cocultivation medium B1 with 2,4-Dichlorophenoxyacetic acid (2,4-D) (0.05 mg/L). After two days on medium B1, explants were moved to post-cultivation medium C with zeatin (2 mg/L) and without indole-3-acetic acid (IAA). After three days on medium C, the explants were transferred to shoot-inducing medium D without IAA for the first two weeks of incubation. After two weeks, media D containing IAA (0.1 mg/L) was used for shoot induction and was renewed every two weeks until successful regeneration occurred. Zeatin was used instead of zeatin riboside for tissue culture media B, C, and D. Shoots were generated from calli and selected for GFP signal with UV light. GFP-positive (transgenic) shoots were moved to root-inducing medium E with IBA (0.25 mg/L) and vancomycin (100 mg/L). Plants with proper root systems were transferred to rockwool and ploidy was determined in leaf samples (Iribov SBW). Diploid shoots were selected for genotypic analysis to confirm the desired mutations.

### 2.5 Selection of mutants and T_1_ segregation

Transgenic, diploid plants were genotyped using Phire Plant Direct PCR (Thermo Scientific). Leaf samples were used to directly amplify the regions of interest with specific primer combinations and the PCR products were Sanger-sequenced to detect mutations. Selected T_0_ mutants were selfed and grown for harvesting T_1_ seeds. In the T_1_ generation, homozygous plants free of the Cas9 transgene construct were selected based on the lack of fluorescence and grown for T_2_ seeds and for further analysis. Primers used for genotyping are listed in **Supplementary Table S4**.

### 2.6 Qualitative and quantitative bud outgrowth analysis

For quantitative and qualitative bud outgrowth analysis, 15 plants per genotype and treatment were grown. Unless stated otherwise, phenotyping was performed at six weeks after sowing (6 WAS), when plants had eight to ten leaves. Leaves (L), buds (B), or axillary shoots (AS) were marked acropetally from L1 to L10, from B1 to B10 and AS1 to AS10, respectively. For quantitative analysis, the lengths of B1 and B4 were measured from the node to the apical meristem. Buds shorter than 0.5 cm were considered inactive and marked as 0, while buds longer than 0.5 cm were considered active and counted as AS. Additionally, the total number of axillary shoots that were longer than 0.5 cm in the axils of the first eight leaves (L1-L8) was counted for each plant. For the decapitated group, plants were decapitated above L4 at four weeks after sowing (4WAS), when plants developed approximately four leaves. The bud quantification was done similarly at 6WAS as in the intact treatment.

For qualitative analysis, buds were classified as dormant, transitioning, or active based on the elongation of the leaf primordia. When shorter than 0.5 cm buds were scored as dormant or transitioning and active when longer. Buds with completely intact leaf primordia were classified as dormant and buds with an elongated leaf primordium as transitioning. Similarly, active buds or axillary shoots were rated according to their vegetative development, where v denotes the growth. The numbers (0, 1, 2, etc.) indicated the number of expanded leaves per shoot. A leaf was considered developed and counted only if it exceeded 2 cm in length. Additionally, the total number of developed leaves and the number of leaves until the first inflorescence were counted for each plant. All phenotyping experiments were independently repeated three times.

### 2.7 Bud harvesting and gene expression analysis

For gene expression analysis, 8–10 plants per genotype were grown and the lowest buds were harvested at 4 pm, 4 WAS. Bud harvesting was done under a binocular microscope and 8–10 buds per genotype were pooled together in acetone. After harvesting, the acetone was removed and bud samples were vacuum infiltrated (Park et al., 2012). Bud samples were stored in -80 until RNA isolation. Bud sampling for both decapitated and intact groups was done in triplicates.

RNA was isolated with the Pico Pure kit (Thermo Scientific) according to the manufacturer’s instructions, followed by Turbo DNase (Invitrogen) treatment to remove any traces of DNA. 120 ng of RNA was used for the cDNA synthesis reaction with iScript (Bio-Rad). Real-time quantitative PCR (RT-qPCR) was performed with SYBR Green Supermix using gene-specific primers (**Supplementary Table S4**), in a CFX6 cycler (Bio-Rad) with the following two-step melting program (3 min 95°C, 40x [15sec 95°C, 1 min 60°C]). As described by Livak and Schimittegen et al., the 2-Delta C(T)) method was used to calculate relative gene expression. *CLATHRIN ADAPTOR COMPLEXES MEDIUM SUBUNIT* (*CAC*, Solyc08g006960) and *EXPRESSED SEQUENCE* (*EXPRESSED*, Solyc07g025390) (González-Aguilera et al., 2016) were selected as reference genes to obtain normalized values. Wild-type intact values were always selected as the calibrator and shown in the figures. All experiments were done with three biological replicates.

### 2.8 Cloning baits for yeast one-hybrid screen and autoactivation tests

Tomato genomic DNA was used to individually amplify each *CR* bait with *CR*-specific primers containing Gateway attB overhangs (**Supplementary Table S4**). The amplified sequences were cloned into the donor vector pDONR221 (Invitrogen) through a BP recombination. An LR recombination was conducted between the entry clone containing *CR* bait sequence and the destination vector pAbai (Takara Bio). Following the small-scale transformation method to integrate the plasmid into the yeast genome, the *CR* bait vectors were separately transformed into a pJ69-4A strain (de Folter and Immink, 2011). The transformed clones were screened with an autoactivation test on the growth medium containing SD-U with different Aureobasidin A (AbA) concentrations (100-, 150-, 200- and 500-mM ng/ul AbA). Clones displaying minimal autoactivation rate were selected for the transformation of the cDNA library.

### 2.9 Generating tomato axillary bud cDNA library

All axillary buds were sampled when plants had two to five expanded leaves. Sampling was conducted under a stereoscope with axillary buds fixated in acetone and subsequently vacuum infiltrated (Park et al., 2012). The Stratec (Qiagen) kit was used according to the manufacturer’s instructions for RNA isolation. DNase treatment was carried out to eliminate any remaining DNA traces with the Turbo DNase kit (Thermo Fischer Scientific). Pure RNA was used to make a primary cDNA library in pDONR201 (Invitrogen) using the CloneMiner II kit (ThermoFischer Scientific) according to the manufacturer’s instructions. To obtain the axillary bud-specific prey library, the LR reaction was performed between the primary library and pDEST22 (Invitrogen).

### 2.10 Y1H library transformations

For library transformations, the pJ69-4A strain containing individual bait constructs was transformed with the prey cDNA library following the large-scale transformation method (de Folter and Immink, 2011). The transformed yeast suspension was incubated on SD-WU media with AbA (125 ng/ul) at 20°C for seven days. Individually growing yeast colonies were resuspended in MQ and spotted on a fresh selection media. These yeast clones were then genotyped using Phire Plant Direct PCR (Thermo Fischer Scientific). The amplicons were Sanger-sequenced with plasmid-specific primers (**Supplementary Table S4**). Finally, the identity of the amplicons was determined through a BLASTN search in the SolGenomics database (https://solgenomics.net/). For those genes not yet functionally characterized in tomato, we examined Arabidopsis homologs by conducting a BLASTp search followed by phylogenetic analysis.

Only transcription factors were selected for further analysis. Following the manufacturer’s instructions, the pDEST22 plasmids of these positive hits were isolated from yeast cells using the Zymo plasmid purification kit (Zymo research). These plasmids were transformed into *E. coli* and Sanger-sequenced for confirmation. The isolated plasmids were then transformed into yeast cells containing the respective *CR* bait to confirm binding. The selection was performed at the same AbA concentration and conditions as in the initial library screen.

### 2.11 Data analysis

Phenotypic experiments were conducted with randomized plant blocks, creating a distinct microenvironment for each genotype. Statistical analysis was performed using the SPSS package to assess the differences within each dataset. Oneway ANOVA was applied to identify significant variations between the means. *Post-hoc* tests, including the least significant difference (LSD) with a significance threshold of *P*-value < 0.05 and *P*-value < 0.01, as well as the Duncan test with a significance threshold of *P*-value < 0.05 were applied to validate statistical differences among different genotypes.

## 3 Results

### 3.1 *SlBRC1B* contains four highly conserved regions in the five kb upstream sequence

Conserved regions (*CR*s) in the upstream sequences of open reading frames are expected to harbor crucial elements for expression regulation, such as *cis*-regulatory elements (CREs). We investigated *CR*s across evolutionary lineages by applying phylogenetic footprinting to sequences of *BRC1-like* genes from various plant species. Initially, we identified closely related *BRC1B* proteins using BLASTp where the first hits clustered as BRC1B and BRC1A of Solanaceae species (**Supplementary Table S1**, **Supplementary Figure S1A**). In Solanaceae, *BRC1A* and *BRC1B* form two subclades originating from a duplication event. In the case of the cultivated tomato, *SlBRC1B* exhibits higher expression in the axillary buds than *SlBRC1A* (Martín-Trillo et al., 2011). However, this study is inconclusive about whether SlBRC1B alone or both paralogs are involved in bud dormancy regulation, underscoring the relevance of the *BRC1A* subclade for further investigation. Although BLASTp highlighted only very closely related *BRC1/TB1* genes, homologous genes have been extensively characterized in other angiosperm species such as in Arabidopsis, maize, and to some extent in cucumber (Aguilar-Martínez et al., 2007; Shen et al., 2019; Studer et al., 2011). All these genes are described as *BRC1/TB1*-clade members (Parapunova et al., 2014; Uberti Manassero et al., 2013). To explore the orthologous relationships among the *BRC1B*-, *BRC1A*-subclade, and *BRC1/TB1* genes, we performed *in silico* synteny analysis. As expected, the gene order in the *BRC1B* subclade showed high synteny among Solanaceae and the genomic organization varied with increasing evolutionary distance (**Supplementary Figure S1B**). Consequently, we selected closely related Solanum *BRC1B*- and *BRC1A*-subclade genes along with distantly related *BRC1/TB1* genes (**Supplementary Table S1**, **Supplementary Figure S1A**) for phylogenetic analysis.

**Figure 1:**
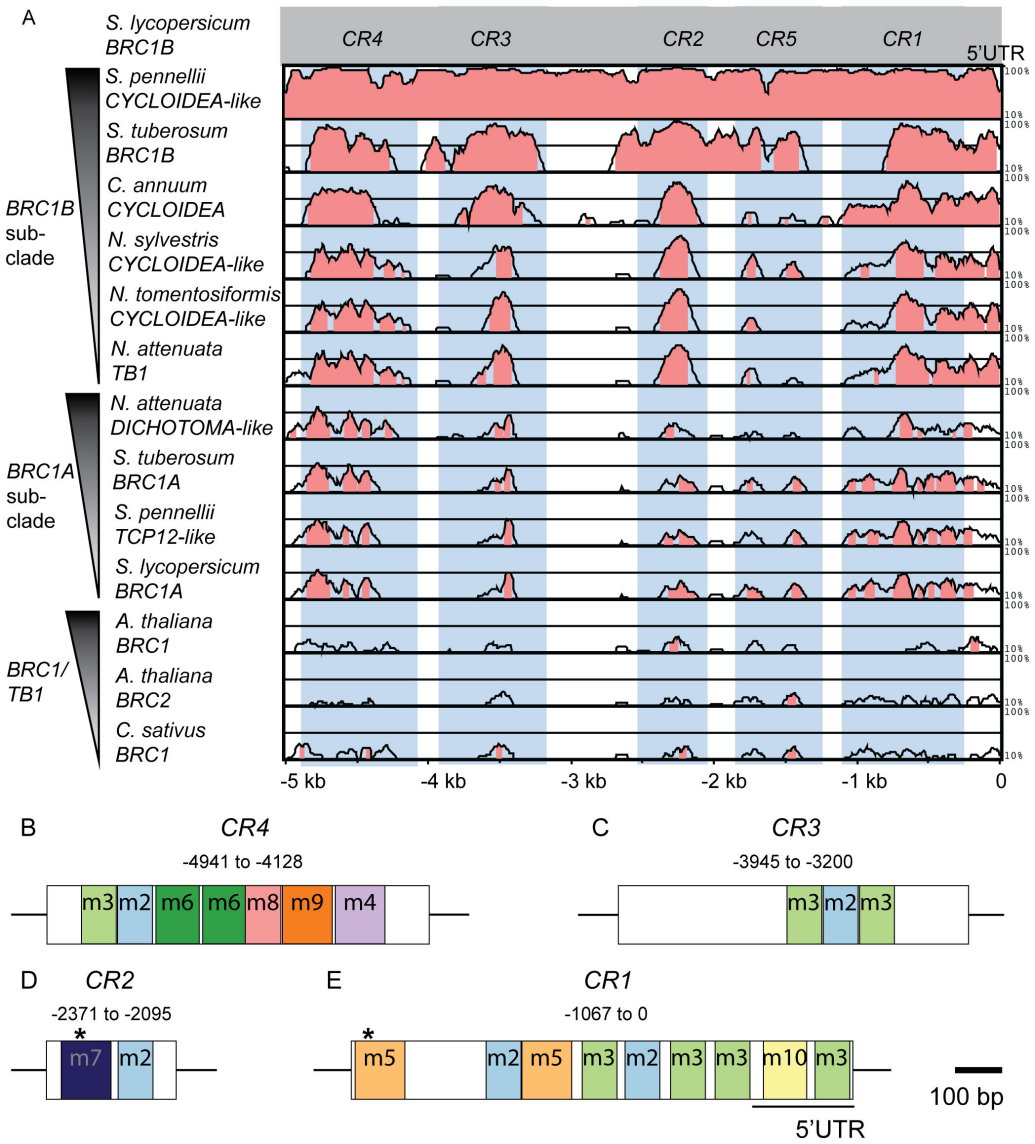
Identification of conserved and putative regulatory regions in the tomato *BRC1B* locus through conservation analysis. **(A)** Five kb upstream sequences relative to the translation start site (ATG) were retrieved from each 14 *BRC1/TB1* homologs for mVISTA analysis (https://genome.lbl.gov/vista/mvista/submit.shtml). The sequences are ordered in the y-axis based on their evolutionary distance to *SlBRC1B*. The LAGAN algorithm was used to identify homologous regions within 50 bp intervals with a significance threshold set at 0.05. The y-axis indicates homologous resemblance, starting at 10% identity. Homologous sequences showing a minimum of 40% identity are highlighted in pink. The positions of conserved regions (*CR*s) are indicated relative to the translation start site of *SlBRC1B* as marked on the x-axis. Blue rectangles highlight each identified *CR*. **(B-E)** Characterization of evolutionarily conserved motif sites in *SlBRC1B CR*s through MEME analysis (https://meme-suite.org/meme/tools/meme). Colored boxes represent distinctive identified motifs (m) residing in *CR4***(B)**, *CR3***(C)**, *CR2***(D)** and *CR1***(E)**. The position of each *CR* is indicated relative to the translation start site, and a scale bar representing 100 bp is provided as a reference for their sizes. Asterisks correspond to binding sites of BZR1 and HY5 in *CR1* and *CR2*, respectively (Dong et al., 2023; Xia et al., 2021). *CR*, conserved region; m, motif.

Phylogenetic footprinting was performed using the putative regulatory sequences of the selected *BRC1* sequences. To compare the five kb upstream sequence of *BRC1A-*, *BRC1B*- subclades and more distantly related *BRC1* genes, we applied relaxed mVISTA criteria requiring a minimum of 40% homology within a 50 bp stretch. This approach enabled the detection of putative regulatory elements in more divergent upstream sequences and revealed five conserved non-coding regions (*CR1*-*CR5*) (**Figure 1A**). These *CR*s showed the highest conservation among the *BRC1B* subclade, followed by *BRC1A* and lower conservation among more distantly related *BRC1* genes. To validate the robustness of these *CR*s, particularly within more closely related species where higher conservation is expected, we reanalyzed the *BRC1A-* and *BRC1B-*subclades in Solanaceae with more stringent, default parameters. This analysis confirmed the presence of four *CR*s (*CR1*-*CR4*) in the *BRC1B* subclade genes with homology exceeding 40% (**Supplementary Figure S2A**). The *BRC1A* subclade, on the other hand, showed weak conservation with these stringent criteria. Next, we compared the five kb downstream sequences between the selected *BRC1* orthologs using the same approach and default parameters. This analysis revealed almost no conservation even within the *BRC1B* subclade, suggesting that downstream regions of *BRC1/TB1* genes contain no or more species-specific elements (**Supplementary Figure S2B**). In contrast, *CR1*-*CR4* exhibited strong conservation across the tested angiosperm species, even under stringent criteria, highlighting them as candidates for important regulatory elements (**Figure 1A**, **Supplementary Figure S2A**). Although *CR1* partially overlaps with the 5′UTR, no uORF or peptide-coding potential was identified within the transcribed portion, supporting its putative role as a non-coding regulatory sequence. Therefore, we selected these four regions for further analysis.

As a next step, five kb upstream regions of *SlBRC1B* homologous sequences were investigated for evolutionarily conserved motifs using Multiple Expectation Maximizations for Motif Elicitation (MEME) (Bailey et al., 2015). Using MEME, we identified 10 significant motifs (m1-m10) across species (**Figures 1B-E**; **Supplementary Figure S3**, **Supplementary Table S2**). While motif m1 was absent in *SlBRC1B*, m2-m10 were all present. Notably, the identified motifs overlap with the previously defined CRs, showing that the CRs indeed contain important conserved regulatory sequences and suggesting that the conserved portions in the CRs that do not overlap with known motifs contain yet unknown additional regulatory sequences. To further characterize each motif, we investigated the presence of putative transcription factor (TF) family consensus binding sites by performing a TOMTOM search against JASPAR database (Fornes et al., 2020). This analysis revealed that various TF consensus binding sites are present in multiple *CR*s (**Supplementary Table S2**), potentially enabling redundant and complex regulation of the *SlBRC1B* promoter by identical or multiple TFs from the same family.

### 3.2 *SlBRC1B CR1*, *CR2*, *CR3* and *CR4* mutants show altered bud outgrowth

**Figure 2:**
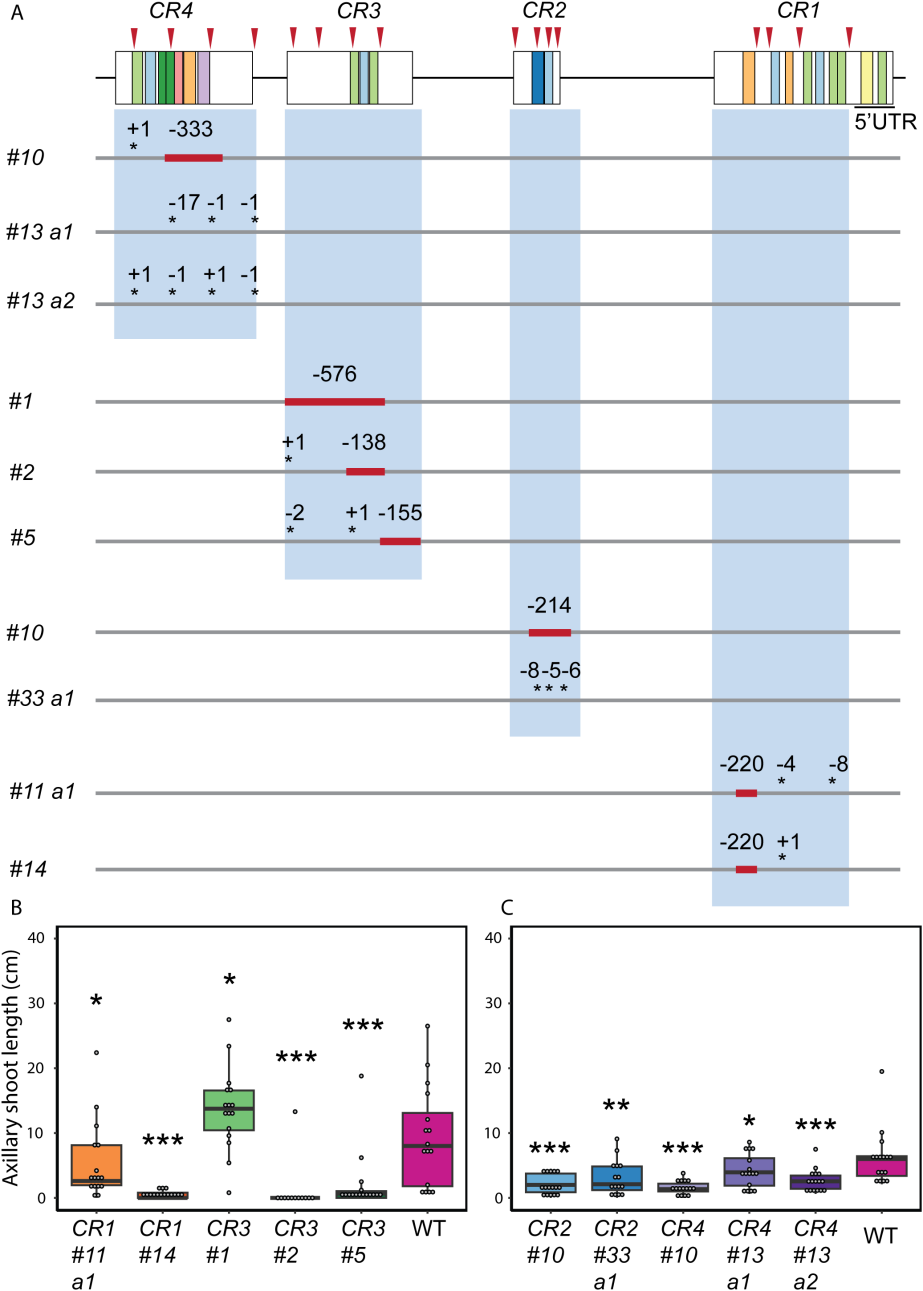
CRISPR alleles of *SlBRC1B* promoter from targeting *CR*s in five kb upstream for bud outgrowth. **(A)** Representation of *SlBRC1B* with identified MEME motif sites (https://meme-suite.org/meme/tools/meme) shown as colored boxes. Each *CR* was targeted with four sgRNAs (red arrowheads) for CRISPR/Cas9 mutagenesis. The red blocks indicate deleted segments, and the asterisks indicate INDELs. Deletions and insertions are represented by a dash (-) and a plus (+), respectively, followed by the number indicating their length. a1, a2: two alleles derived from the same T_0_ mother plant. **(B, C)** The length of axillary shoot of *CR1* and *CR3***(B)** or *CR2* and *CR4* mutants **(C)** (*N* = 12-15). Statistical significance is determined using one-way ANOVA with LSD *post-hoc* tests. Significance levels of *p* < 0.05, *p* < 0.01 and *p* < 0.001 are indicated by single, double and triple asterisks respectively.

Since *CR1* to *CR4* exhibited the highest conservation throughout the Solanaceae family (**Figure 1**), we focused on these regions to understand their potential role in *SlBRC1B* regulation and functioning *in vivo*. We targeted each *CR* individually for deletion by multiplexed CRISPR/Cas9-mutagenesis with four sgRNAs per region. The mutagenesis approach resulted in large deletions, in some cases removing nearly the entire region, or in small indels at the respective sgRNA positions (**Figure 2A**, **Supplementary Figure S4**). Subsequently, ten unique mutant alleles were selected for further analyses. Homozygous mutants were identified by genotyping in the next generation after selfing and screened to assess the potential effects of the mutations on bud growth at six weeks after sowing (6WAS). We measured the length of bud1, because it exhibited the most substantial outgrowth compared to other buds in a previous study (Martín-Trillo et al., 2011). In this way, the bud outgrowth of each *CR* mutant was compared with that of the wild-type in two independent experiments, one with *CR1* and *CR3* mutants and another with *CR2* and *CR4* mutants. Wild-type control plants were included in each experiment to compensate for varying environmental conditions that might affect the bud outgrowth. Strikingly, all *CR* mutants except *CR3 #1* exhibited significantly decreased axillary shoot length (**Figures 2B, C**; **Supplementary Figure S5**). Moreover, some *CR* mutants such as *CR1 #14*, *CR2 #10*, *CR3 #2*, and *CR4 #10* showed almost no outgrowth. Together, these results indicate that several *CR* mutations inhibited bud outgrowth.

**Figure 3:**
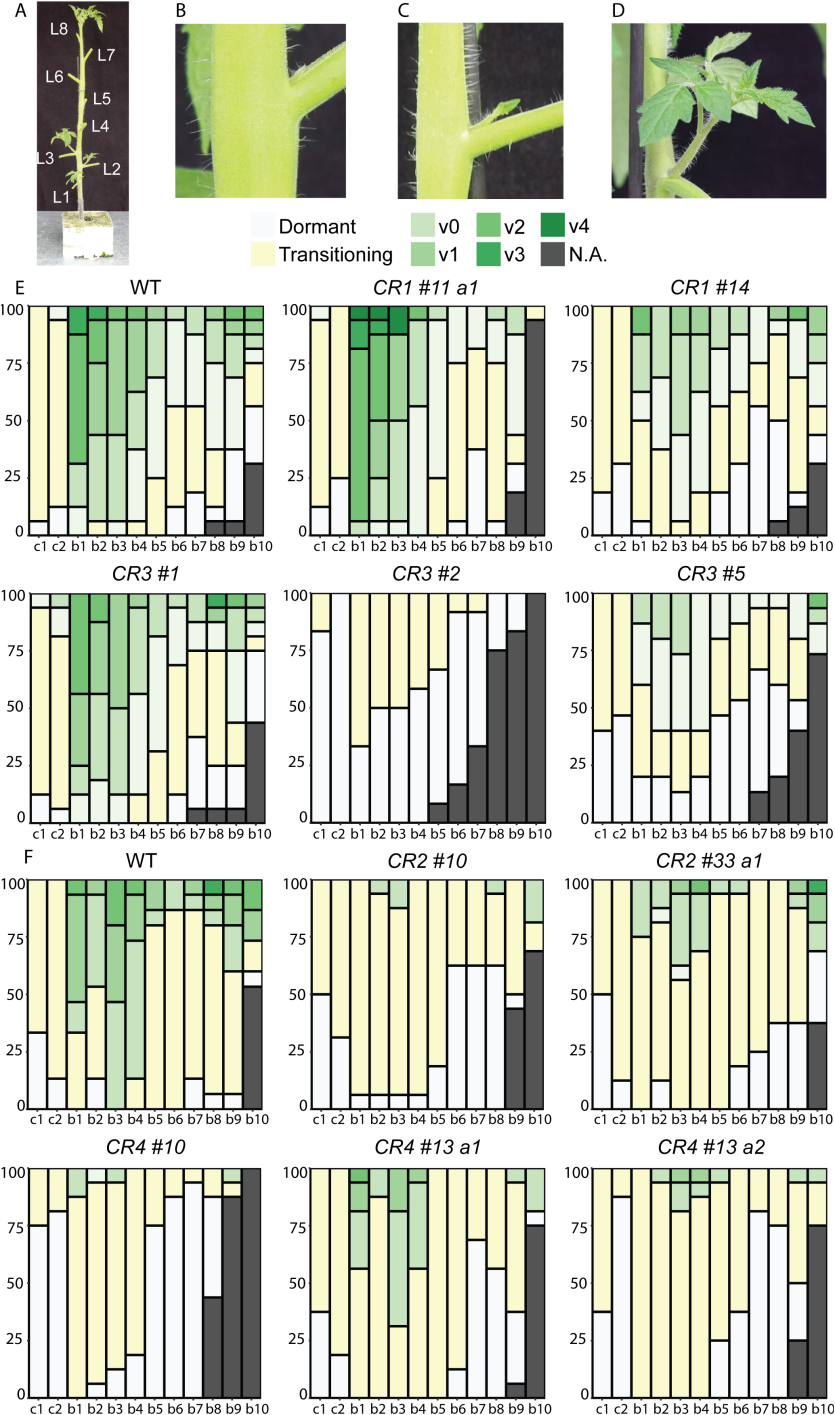
Detailed characterization of bud outgrowth in *SlBRC1B CR* mutants. **(A)** Tomato plant with eight expanded leaves, at 6 WAS, showing the stage used for phenotyping of branching for both wild-type and mutant plants. Axillary buds and leaves were counted acropetally, and leaves were removed for clarity. **(B-D)** Bud development stages: a dormant bud with intact primordia **(B)**, a transitioning bud with an elongated primordium **(C)**, an active bud (axillary shoot longer than 0.5 cm) **(D)**. Active buds were rated by vegetative development, where “v” stands for vegetative growth, with numbers (0, 1, 2, etc.) indicating the number of expanded leaves per shoot. **(E, F)** Characterization of bud or axillary shoot development in *CR1* and *CR3***(E)**, or *CR2* and *CR4* mutants **(F)** (*N* = 15). Stacked percentage plot showing bud outgrowth status across axil positions (cotyledons to L8). Each column represents 15 plants; the y-axis shows percentage (0–100%) and the x-axis indicates axil position. Color codes: Gray and yellow for the axillary buds (< 0.5 cm) and shades of green for the axillary shoots (> 0.5 cm). Phenotyping experiments had three independent repeats performed at different times with similar results, simultaneously for *CR1* and *CR3*, or *CR2* and *CR4*, respectively. WAS: weeks after sowing, b: bud, L: expanded leaf, c: cotyledon, a1/2: allele 1/2, v: vegetative, N.A., not applicable.

Given that *CR* mutations altered bud growth, we next comprehensively characterized the development of all visible buds (b) in *CR* mutants. Following the same experimental setup with two independent experiments, buds were scored as dormant and transitioning when shorter than 0.5 cm or active when longer (**Figures 3A-D**). Active buds were then scored for the number of developed leaves they had. Additionally, although each bud (b1 to b8) was scored individually, we refer to b1-b4 as basal buds, located at the lower nodes, and b5-b8 as upper buds, positioned higher on the stem, to aid interpretation. Several *CR* mutants exhibited significantly altered bud development (**Figures 3E, F**; **Supplementary Figure S5**). For instance, the basal leaf axils of all *CR* mutants, except *CR1 #11 a1* and *CR3 #1*, mostly contained inactive buds, or buds at the earliest stages of shoot development. This phenotype was significantly different from the wild-type, which mostly had axillary shoots in the later stage of development. Interestingly, *CR2 #10*, *CR3 #2*, *CR4 #10*, and *CR4 #13 a2* mutations had stronger inhibitory effects on bud development than other mutations. Almost all buds were at the transitioning stage, and in these mutants, dormant upper buds were more frequent compared to other mutants and wild-type plants. Moreover, *CR3 #2* had no buds in the active bud class and developed fewer expanded leaves than wild-type, indicating strongly delayed outgrowth and retarded shoot growth (**Figure 3E**). Overall, these results indicate that *CR* mutations affected both bud growth and development, suggesting that critical regulatory regions located upstream of *SlBRC1B* are disrupted.

### 3.3 *SlBRC1B CR* mutants show minimal changes in other developmental aspects of the primary shoot

**Figure 4:**
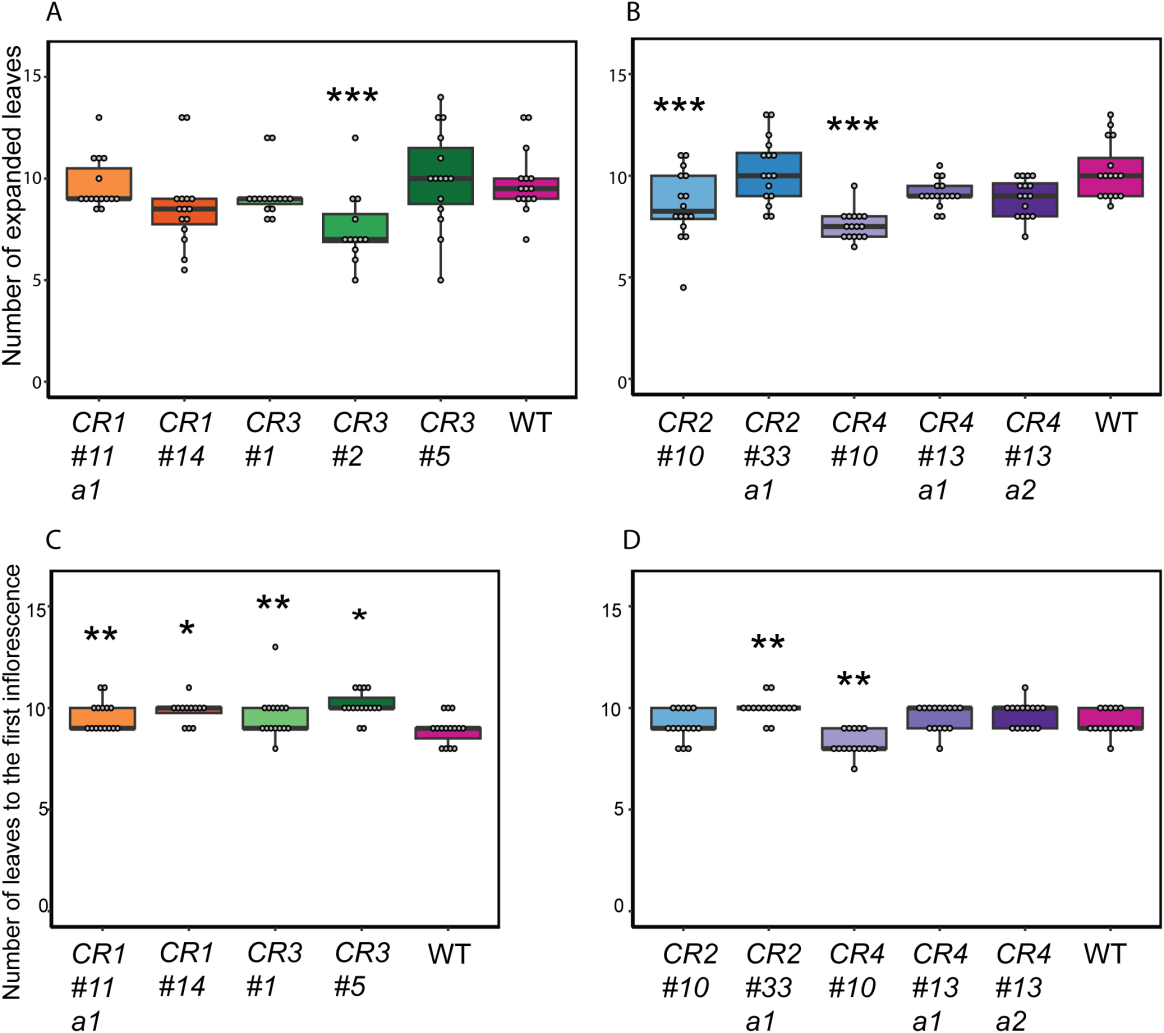
Impact of *SlBRC1B CR* mutations on other developmental aspects of the primary shoot. **(A, B)** The number of expanded leaves of *CR1* and *CR3***(A)**, *CR2* and *CR4***(B)** mutants compared to wild-type (WT) tomato plants at 6 WAS. **(C, D)** Flowering time of *CR* mutants. Leaves up to the first inflorescence were counted as a proxy for flowering time for *CR1* and *CR3***(C)**, or *CR2* and *CR4***(D)** mutants at 6 WAS. *CR3 #2* mutants were excluded from the flowering time analysis, as none reached the flowering stage at 6 WAS, due to delays in the primary shoot development. One-way ANOVA is applied to determine statistical significance, followed by an LSD *post-hoc* test. Significance levels of *p* < 0.05, *p* < 0.01, and *p* < 0.001 are indicated by single, double, and triple asterisks, respectively. a1/2: allele 1/2. WAS: weeks after sowing.

Next, we examined whether mutations in the promoter region affected the development of other tissues or influenced the overall development of the mutants. Previous studies in Arabidopsis indicated that ectopic *BRC1* expression reduces axillary shoot growth and delays the development of other tissues. This also resulted in thinner and shorter stems and, in some cases, the arrest of the shoot apical meristem (Aguilar-Martínez et al., 2007). Moreover, AtBRC1 is shown to interact with florigen FLOWERING LOCUS T (FT) to regulate axillary shoot outgrowth and flowering (Fichtner et al., 2021; Niwa et al., 2013). Given the observed effects of *BRC1* when ectopically expressed, we assessed the number of developed leaves and flowering time at 6WAS as developmental readouts. Only *CR2 #10*, *CR3 #2*, and *CR4 #10* showed significantly fewer expanded leaves compared to wild-type (**Figures 4A, B**). While *CR1 #11 a1*, *CR2 #33 a1*, and *CR3 #1* mutants showed a more pronounced delay in their flowering time, *CR4 #10* flowered significantly earlier than the wild-type (**Figures 4C, D**). Overall, no or mild effects were found and therefore, we concluded that *CR* mutants, except for *CR3 #2* and *CR4 #10*, demonstrated minimal changes in shoot development (**Figure 4**). Given that *CR4* mutants showed different degrees of decreased bud outgrowth with minimal pleiotropic effects and that this promoter region contained multiple putative TF binding sites (**Figures 1B**, **2**, **4**; **Supplementary Table S2**), we selected this region for further analysis.

### 3.4 Potential molecular causes of decreased bud outgrowth in *SlBRC1B CR4* mutants

**Figure 5:**
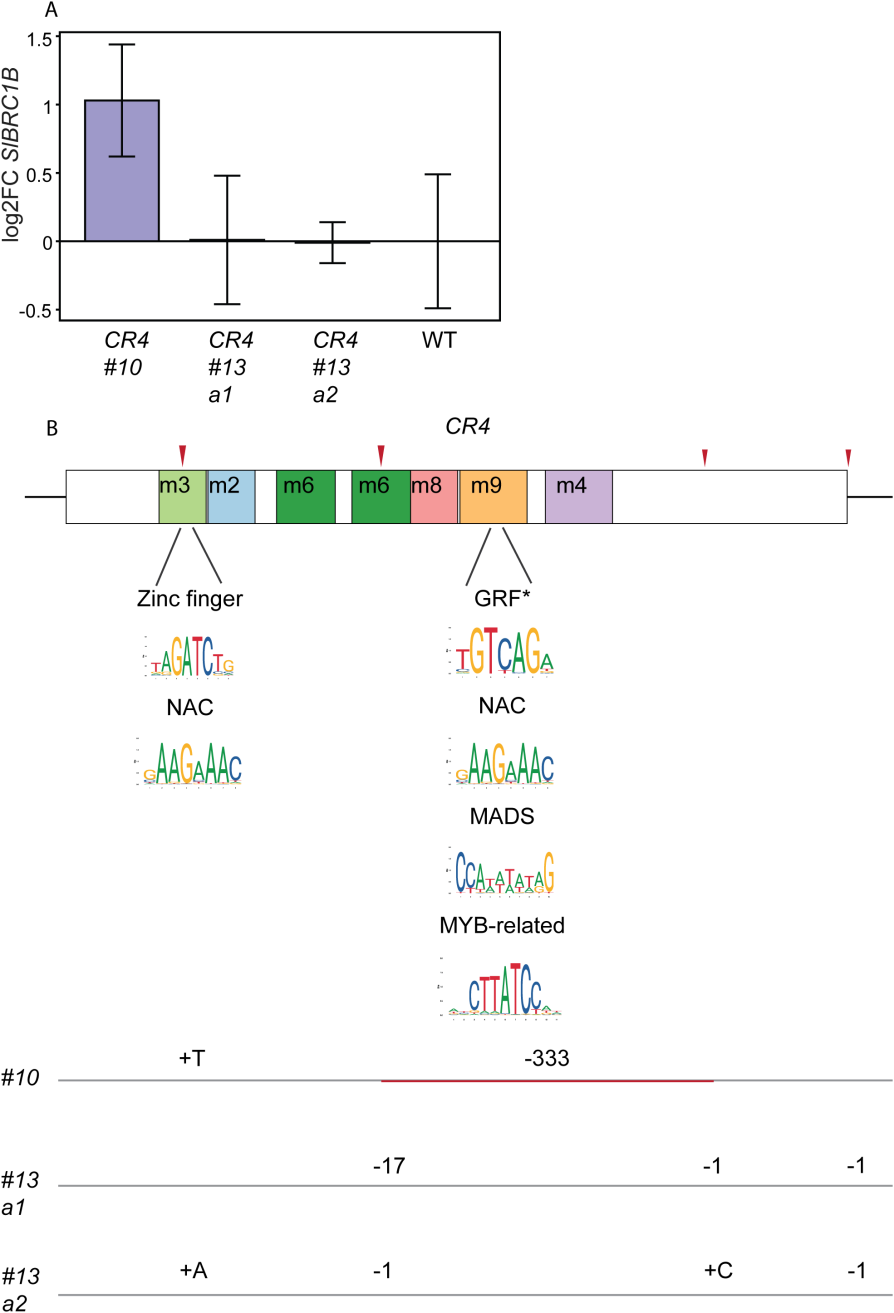
Detailed analysis of *CR4* mutants and their sequences. **(A)***SlBRC1B* mRNA levels were investigated in bud1 of *CR4* mutants and wild-type (WT) by RT-qPCR at 4 WAS. 8–10 buds were pooled for each biological replicate, and three replicates per genotype were used. *SlBRC1B* expression was normalized against reference genes *CLATHRIN ADAPTOR COMPLEXES MEDIUM SUBUNIT* (*CAC*, Solyc08g006960) and *EXPRESSED SEQUENCE* (*EXPRESSED*, Solyc07g025390), and relative expression was calculated against the values of wild-type plants. SEM means were calculated, and t-test (*p* < 0.05) was applied for statistical significance. **(B)***In silico* predicted consensus binding sites corresponding to TFs identified as binding to *CR4* in the Y1H library screens. Lines below *CR4* indicate the locations of the mutations that lead to the removal of the specific TF binding sites. The removed consensus sites are highlighted with a logo beneath the respective TF. Consensus binding sites for each TF, except GRF marked with an asterisk, were identified in multiple motif sites (summarized in **Supplementary Table S3**). WAS: weeks after sowing, a1/2: allele ½, m: motif sites identified by MEME, GRF: GROWTH REGULATING FACTOR.

*CR4* mutants exhibited the strongest reduction in axillary shoot development and growth compared to wild-type and minimal, if any, defects in other organs (**Figures 2C**, **3F**, **4B, D**). These results suggest that critical elements within *CR4* altered the expression level of *SlBRC1B* in the axillary buds without compromising tissue specificity. We focused on *SlBRC1B* expression in bud1, based on the previous study (Martín-Trillo et al., 2011) and our phenotypic observations (**Figures 2B, C**, **3E, F**). We hypothesized that where bud1 of a wild-type plant is released from dormancy, *CR4* mutants could still show increased *SlBRC1B* expression, explaining the extended dormancy phenotype. Bud1 of *CR4* mutants and wild-type were harvested when plants had four expanded leaves (4L) at 4 WAS, followed by RT-qPCR with *SlBRC1B*-specific primers. In most *CR4* mutants, we couldn’t detect a significantly altered *SlBRC1B* expression when compared to the wild type (**Figure 5A**). However, in *CR4 #10*, which had the most severe delay in axillary bud development (**Figures 2C**, **3F**), an almost two-fold increased *SlBRC1B* expression compared to the wild-type was found.

Next, we aimed to identify TFs that can bind to *CR4* by using it as bait in a Yeast one-hybrid (Y1H) screen with a tomato axillary bud cDNA library as prey. Because we were interested in the transcriptional regulation of *BRC1B* during early stages of bud development, we selected plants of different ages with two to five expanded leaves (2L-5L). From these plants, axillary buds were harvested and used to construct a tomato axillary bud cDNA library. Subsequently, this library was transferred into the Y1H GAL4-Activation domain (AD) prey vector and an autoactivation test was performed for the *CR4* bait construct using a concentration range of the selective agent Aurobasidin A. The Y1H screen of the axillary bud cDNA library with the *CR4* baits resulted in multiple hits. The prey (GAL4-AD) vector insertions of these hits were sequenced and analyzed using BLASTN on the Solgenomics database. Among these, we focused only on genes classified as TFs (**Supplementary Table S3**). This resulted in a list of candidate TFs, including members of the GROWTH REGULATING FACTOR (GRF), zinc finger, NAC, MADS and MYB-related families whose binding was confirmed in an independent repetition of the experiment.

Notably, several TF consensus binding sites identified by the *in silico* TOMTOM analyses (**Supplementary Table S2**) corresponded to TFs identified in the Y1H screens (**Supplementary Table S3**) and targeted in the generated CRISPR alleles (**Figure 2A**). For instance, both *CR4 #10* and *CR4 #13 a2* exhibited a mutation in the m3 site, which is predicted to contain binding sites for zinc finger and NAC TFs (**Figure 5B**, **Supplementary Table S3**). The Y1H screens resulted in the identification of members from both TF families. Since these binding sites were removed in the *CR4 #10* and *CR4 #13 a2* mutants, their loss could explain the decreased bud outgrowth. Moreover, in *CR4 #10* mutants both m8 and m9 sites were removed, which contained additional binding sites for zinc finger, GRF, NAC, MADS, and MYB-related TFs. Overall, these results suggest that TFs from the zinc finger, GRF, NAC, MADS, and MYB-related families function as regulators of *SlBRC1B* expression potentially through cooperative interactions.

## 4 Discussion

Endogenous and exogenous cues regulate *BRC1* activity, but the detailed mechanisms underlying its transcriptional regulation are still largely unknown. In this study, we investigated the putative transcriptional *cis* and *trans* regulators of *SlBRC1B* through comparative sequence analysis followed by *in vivo* and *in vitro* approaches. Phylogenetic footprinting with five kb upstream sequences relative to the translation start site of *BRC1/TB1* homologs identified four *CR*s across angiosperm evolution (**Figure 1**). Mutagenesis of four *CR*s resulted in at least one mutant with decreased bud outgrowth for each *CR*. *CR4 #10* and *CR4 #13 a2* showed the biggest decrease in outgrowth compared to the wild-type (**Figures 2**, **3**), yet measurable effects at the transcriptional level were limited (**Figure 5A**). Subsequently, Y1H screens with *CR4* identified zinc finger, GRF, NAC, MADS and MYB-related TFs as potential regulators, and in line, the *CR4 #10* mutant lacked their putative binding sites (**Figure 5B**; **Supplementary Tables S2**, **S3**). Together, this provides insight into the transcriptional control of *SlBRC1B*, its robust regulation and role in axillary bud development.

### 4.1 *SlBRC1B* activity regulation is primarily repressor-dependent

BRC1 functions as a critical signal integrator in axillary buds, responding swiftly to various cues that regulate bud outgrowth (Aguilar-Martínez et al., 2007; González-Grandío et al., 2013; Gonzalez-Grandio et al., 2017; González-Grandío and Cubas, 2014; Martín-Fontecha et al., 2018; van Es et al., 2024). Our results suggest that this rapid response could be facilitated by repressor-dependent regulation because most *SlBRC1B* promoter mutants exhibited decreased bud outgrowth (**Figures 2**, **3**; **Supplementary Figure S5**). Main regulation by repression instead of activation is common in plants and previous studies revealed e.g., that repressor TFs primarily regulate *AUXIN RESPONSE FACTOR 7* (*ARF7*) in Arabidopsis. *AtARF7* features an open chromatin structure, allowing rapid response to endogenous and exogenous cues without reliance on chromatin modifications. Instead, the specific expression pattern of *AtARF7* is maintained by repressor TFs (Truskina et al., 2021). In tomato, a recent study on leaf tissue found that the region five kb upstream of the *SlBRC1B* gene mostly lacked the repressive histone mark H3K27m3 (Lü et al., 2018). Interestingly, despite this open chromatin state, *SlBRC1B* expression remains hardly or not detectable in leaf tissue (Martín-Trillo et al., 2011), suggesting that low *SlBRC1B* expression in each tissue may not necessarily depend on chromatin inaccessibility. It is plausible that repressor-dependent regulation contributes to the inhibition of *SlBRC1B* expression in leaves and a similar mechanism may also operate in tomato axillary buds. However, further studies are needed to determine whether *SlBRC1B* regulation is primarily repressor-dependent and whether its chromatin structure is and remains accessible at different stages of bud development.

### 4.2 *CR4* mutants show minimal changes in overall *SlBRC1B* expression levels

In several promoter mutants with altered axillary bud outgrowth, qRT-PCR did not reveal a significant change in *SlBRC1B* expression, except for *CR4 #10*, which exhibited an almost two-fold increase (**Figure 5A**). Notably, previous studies in Arabidopsis showed that bud activation signals, such as decapitation, downregulate *AtBRC1* expression at most two-fold within 1 to 48 hours after decapitation (Aguilar-Martínez et al., 2007). Similarly, in tomato, decapitation led to a two-fold decrease in *SlBRC1B* expression within 8 hours (Martín-Trillo et al., 2011), differences close to the detection limit of qRT-PCR. Given that *BRC1* expression changes in these situations were relatively small, we concluded that even transient or subtle reductions are sufficient to trigger or hold out bud outgrowth. These observations, together with our results, suggest that *SlBRC1B* expression is regulated within a narrow range. Furthermore, *in situ* studies of axillary buds of both intact Arabidopsis and tomato plants revealed that *BRC1* expression expanded into the vasculature of the buds as leaf primordia elongated (Aguilar-Martínez et al., 2007; Martín-Trillo et al., 2011). Thus, the decreased bud outgrowth of *CR4 #13* mutants may arise from subtle spatiotemporal alterations in *SlBRC1B* expression, where downregulation is delayed or prolonged into later stages of bud development, preventing timely bud activation.

### 4.3 Potential redundancies between *CR*s

*In silico* analysis revealed that the conserved motifs in *CR*s contained binding sites of zinc finger, NAC, MADS, and MYB(-related) TF families (**Supplementary Table S2**). All four *CR*s shared common binding sites for the same TF families, suggesting redundant regulation of *SlBRC1B* by several *CR*s. Additionally, our experiments mutating individual *CR*s demonstrated only partial inhibition of bud outgrowth, indicating that combining the most interesting mutations might be necessary to understand their effects on bud outgrowth fully and to obtain more profound effects (**Figures 2**, **3**). Although most *CR* mutations decreased bud outgrowth to some extent, the variability in phenotypic strength implies that these regions likely contain not only repressive but also activating elements. Given that transcriptional regulation is shaped by complex interplay between multiple transcription factors (Kaufmann et al., 2010; Reiter et al., 2017; Weingarten-Gabbay and Segal, 2014), partial redundancy among *CR*s may buffer the effect of individual mutations.

The existing model, where BRC1 is proposed to regulate the bud activation threshold (Seale et al., 2017), points to the potential value of investigating the cumulative effects of *CR* mutations on outgrowth. However, a recent investigation of the upstream and downstream regulatory regions of the highly conserved *CLAVATA3* (*CLV3*) in Arabidopsis and tomato indicates that this may prove challenging (Ciren et al., 2024). Nevertheless, our results indicated potentially redundant *SlBRC1B* regulation via *CR1*, *CR2*, *CR3*, and *CR4* and the importance of specific *CR* modifications (**Figures 1B-E**, **2**, **3**; **Supplementary Table S2**). This information could be used to finetune *SlBRC1B* activity in the axillary buds.

### 4.4 Targeting *CR*s to improve plant architecture

Here we demonstrated that targeting evolutionary conserved non-coding regions of *SlBRC1B* significantly affected bud outgrowth. This supports the approach as a viable strategy for identifying regulatory regions and finetuning developmental traits. The potential of this approach was previously shown to inflorescence branching and fruit size in tomato (Rodríguez-Leal et al., 2017). In line with this concept, various molecular analyses revealed that improvements of numerous traits in conventional breeding programs, are due to the selection of favored promoter variants resulting in the optimal expression level of the downstream gene (Meyer and Purugganan, 2013; Swinnen et al., 2016). For example, increased fruit size in tomato was caused by mutations in CREs of *WUSCHEL* (*WUS*) and by a large inversion affecting the upstream regulatory region of *CLV3*, two genes involved in regulating floral meristem size (Muños et al., 2011; Rodríguez-Leal et al., 2017). In maize, the transposon insertion *hopscotch* in the upstream sequence of *TB1* led to the higher expressing variant *tb1*, which is responsible for the striking differences in plant architecture between the cultivated species and its branched wild ancestor, teosinte (Studer et al., 2011). Identification and targeted modification of these CREs, therefore, can result in varied expressions of the gene of interest, enabling the possibility of deciphering the function of TFs with minimal pleiotropic consequences in the plant body (Rodríguez-Leal et al., 2017; Wittkopp and Kalay, 2012). Hence, molecular analysis emphasizes that creating mutations in CREs rather than knocking out the gene itself is a proven strategy for improving traits.

## Data Availability

The datasets presented in this study can be found in online repositories. The names of the repository/repositories and accession number(s) can be found in the article/**Supplementary Material**.
