# Supplementary material for: Conserved regions upstream of BRC1B regulate bud dormancy in tomato
Source: Front Plant Sci. 2025 Nov 20;16:1702139. doi: 10.3389/fpls.2025.1702139 (PMC12675364; doi:10.3389/fpls.2025.1702139)
Supplement: Supplementary file 1 [file DataSheet1.docx]

**Supplementary Material**

**1 Supplementary Figures and Tables**


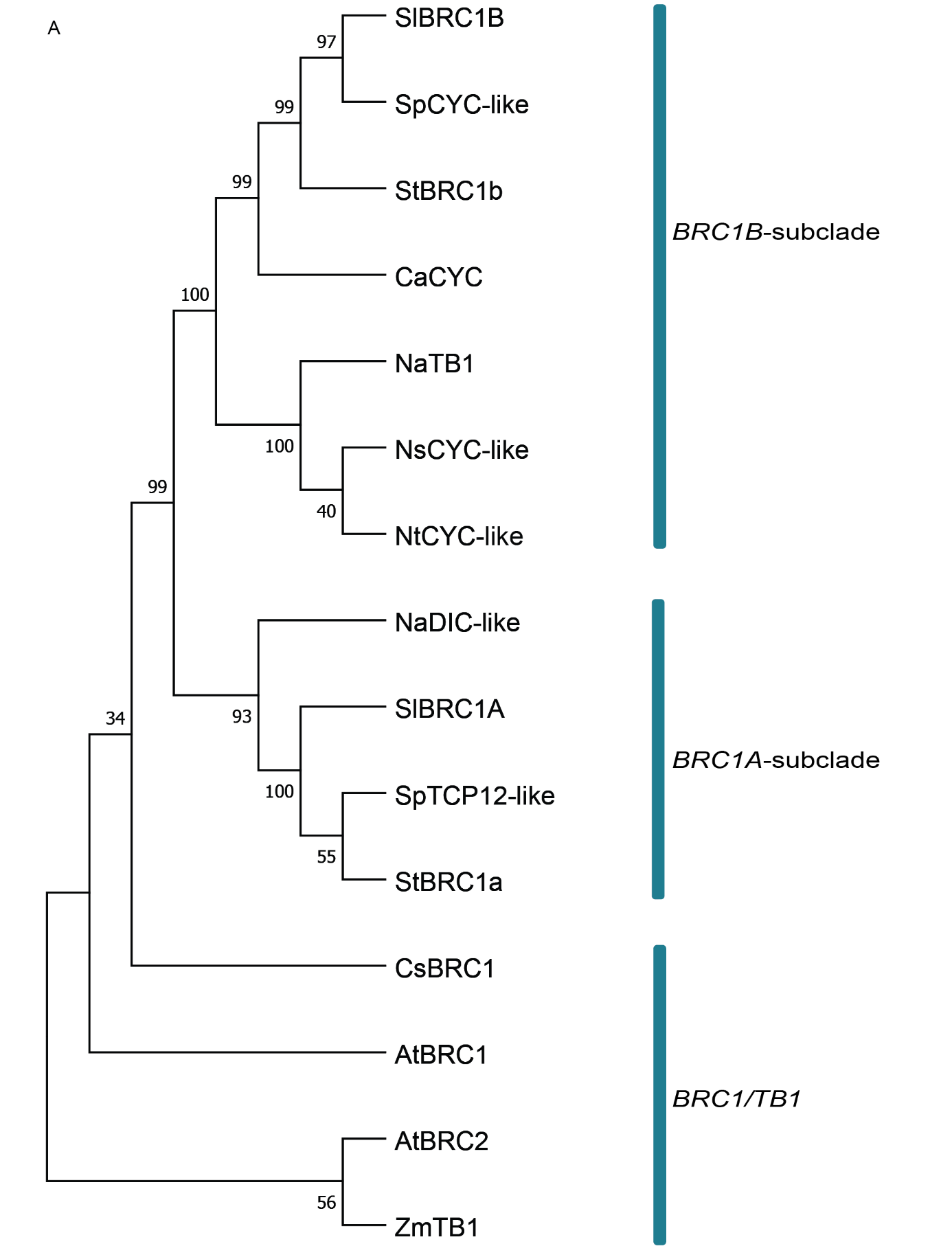


**
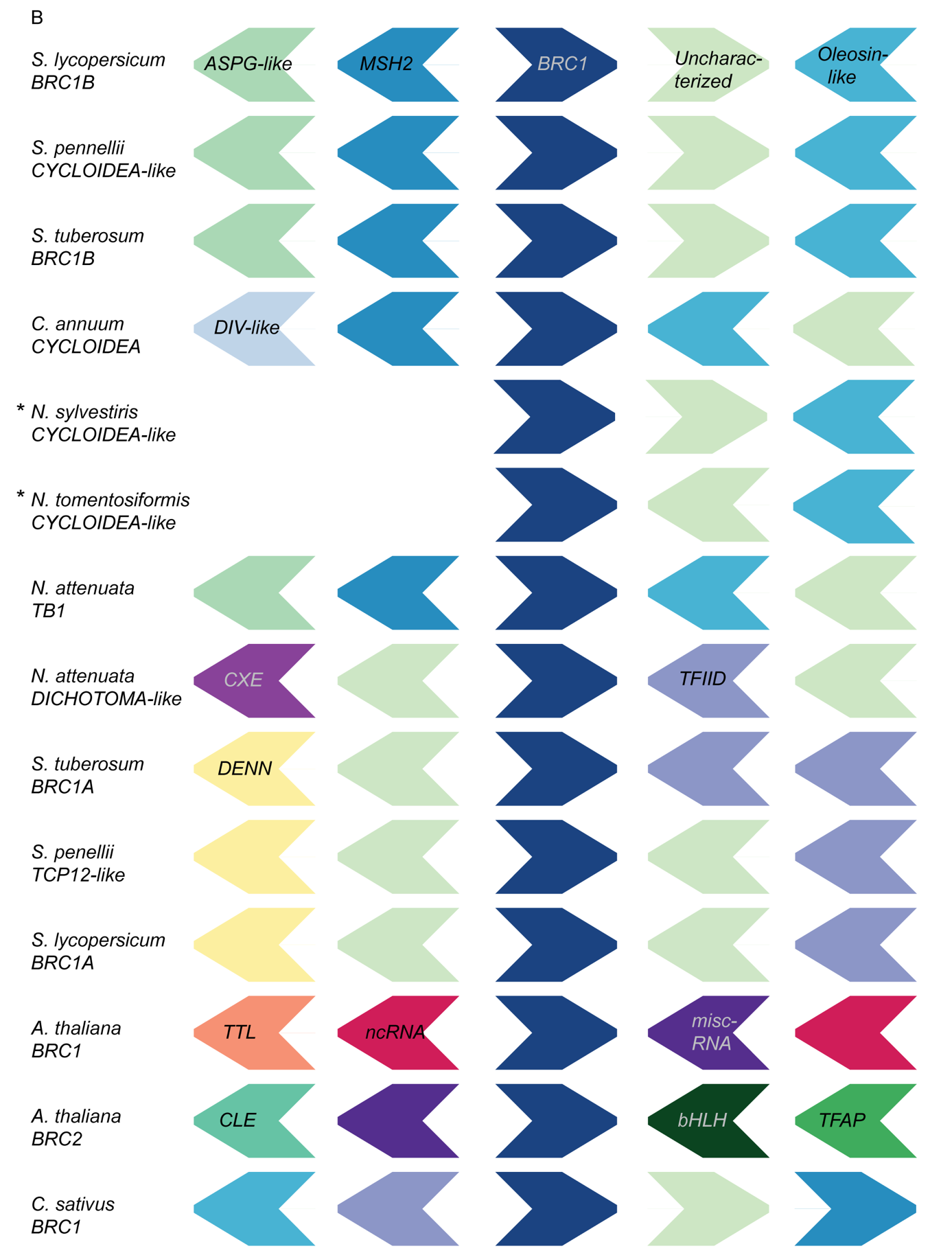
 Supplementary Figure S1** Identification of closely related SlBRC1B homologs across different species. **(A)** Phylogenetic relationship of closely related *SlBRC1B* genes. The phylogenetic tree was constructed using the coding sequences (CDS) of *BRC1B*- and *BRC1A*- subclade genes of Solanaceae species and *BRC1/TB1* genes from Arabidopsis, cucumber and maize. A neighbor-end joining (NJ) tree with 1000 bootstrap replicates was used. **(B)** Genomic organization of *SlBRC1B* homologs from Solanaceae along with Arabidopsis, and cucumber. The two closest neighboring genes both upstream and downstream of *SlBRC1B* homologs are shown. Each gene family is represented by a different color and the direction of the arrowhead indicates their orientation. In the case of the *CYCLOIDEA-like* gene of *N. sylvestris* and *tomentosiformis*, marked with an asterisk, the upstream neighboring genes are not shown because *BRC1* is located at the beginning of the chromosome. *ASPG-LIKE: ASPARTIC PROTEASE IN GUARD CELL-LIKE*, *MSH2: MUTS HOMOLOG 2*, *DIV: DIVARICATA*, *DENN*: *DIFFERENTIALLY EXPRESSED IN NORMAL AND NEOPLASTIC CELLS*, *CXE: CARBOXYLESTERASE*, *TTL: TUBULIN TYROSINE LIGASE*, *CLE: CLAVATA3/ESR-RELATED 12*, *TFAP: TRANSMEMBRANE FRAGILE-X-F-ASSOCIATED PROTEIN*, *CYC: CYCLOIDEA*, *DIC: DICHOTOMA*.


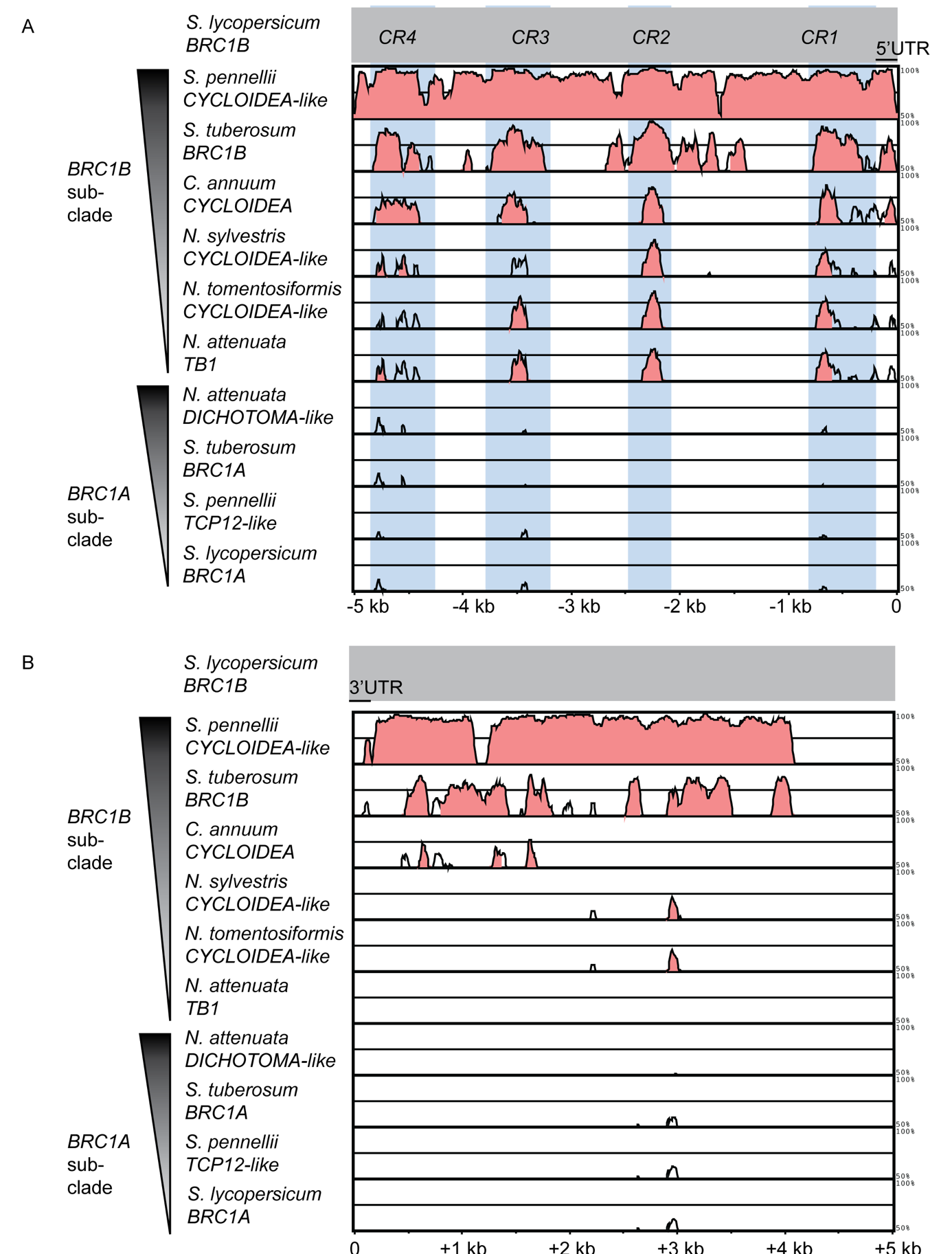


**Supplementary Figure S2** mVISTA conservation analysis of selected *SlBRC1B* homologs. Five kb upstream **(A)** and downstream **(B)** of *SlBRC1B* homologous sequences. Homologous regions were identified using the LAGAN algorithm at 50 (upper panel) or 100 bp (lower panel) intervals with a significance value of 0.05. Pink highlights denote regions with over 40% homology. The y-axis displays the percentage of homology and sequences are ordered based on their evolutionary distance from *SlBRC1B*. Conserved regions in upstream sequences are indicated on the x-axis with blue rectangles and are numbered relative to the translation start site of *SlBRC1B*. *CR*: conserved region.


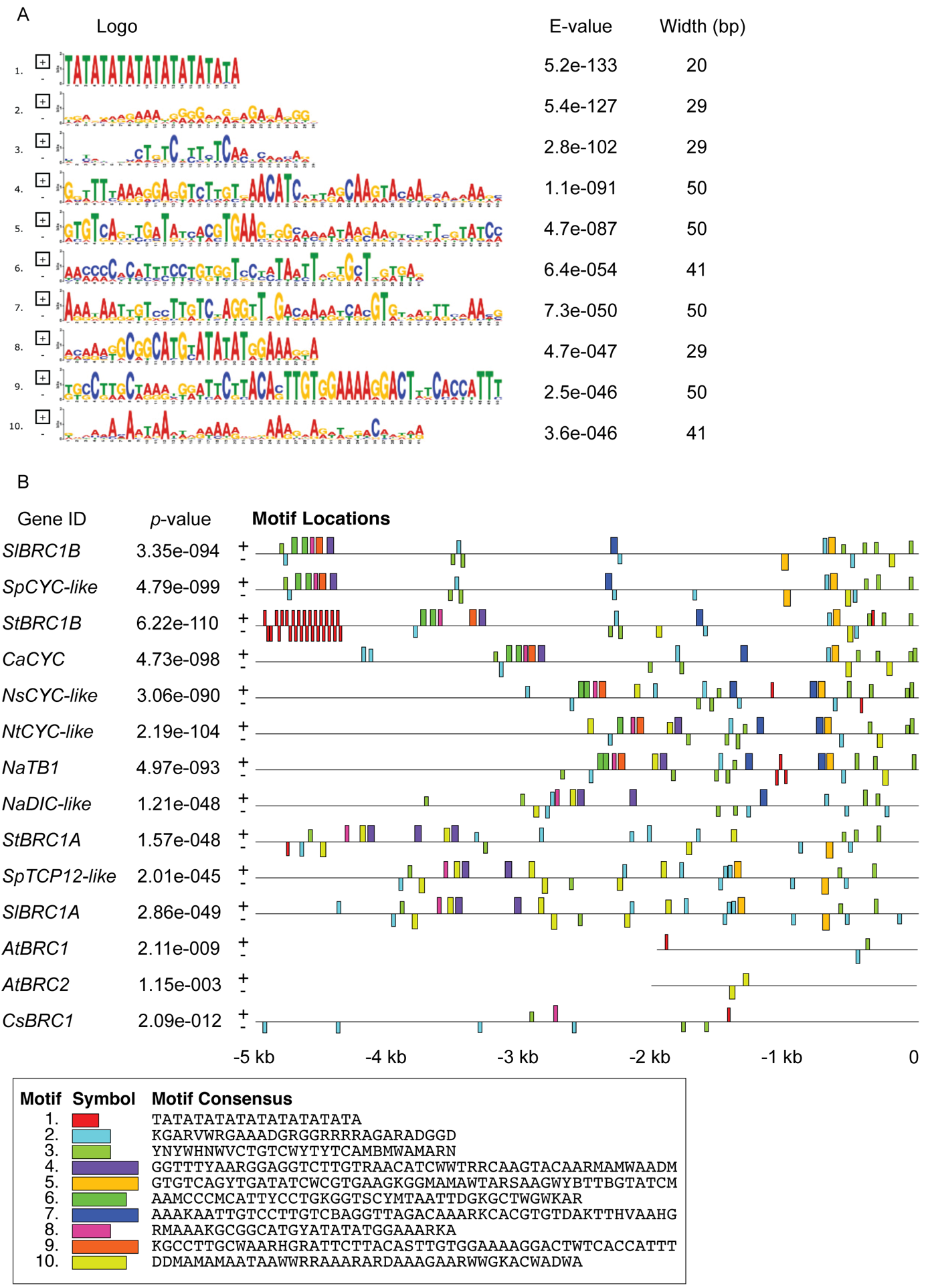


**Supplementary Figure S3** Identification of MEME motif sites in 5 kb upstream of *SlBRC1B* homologs. **(A)** The top 10 significantly identified MEME motifs are displayed with their corresponding sequence logos and widths. These motifs are ordered according to their significant E-value on the y-axis. **(B)** Locations of 10 motifs were investigated 5 kb upstream for all *SlBRC1B* homologs, except for Arabidopsis, where they were examined 2 kb upstream, up to the adjacent upstream gene. The orientation of each motif is indicated with plus (+) for the forward and minus (-) for the reverse strand. Homologs are ordered on the y-axis according to their evolutionary distance to S*lBRC1B*. A distinct color represents each motif. The consensus sites of the identified motifs are indicated in the box, each in its respective color. CYC: CYCLOIDEA, DIC: DICHOTOMA.

**
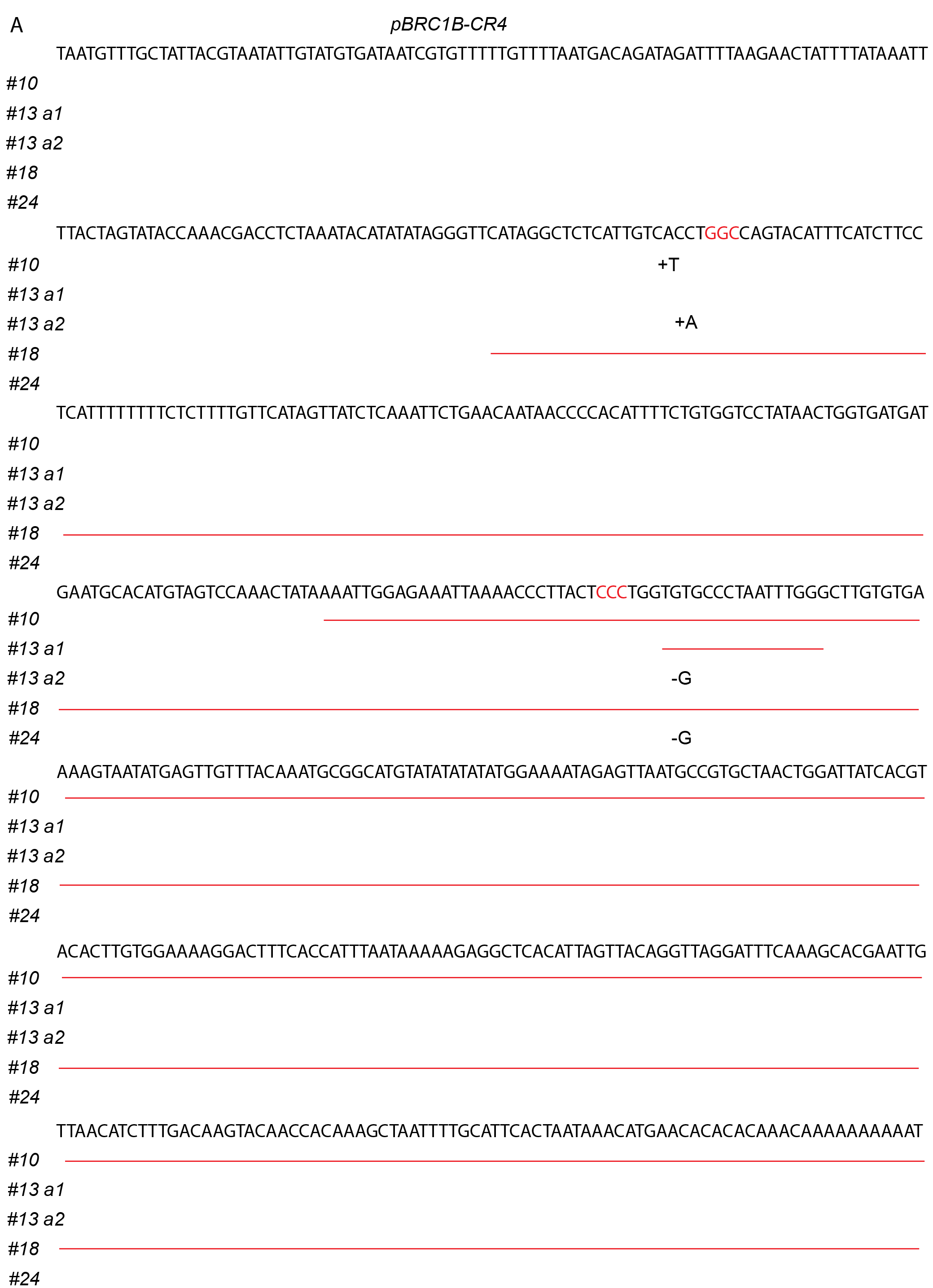
**

**
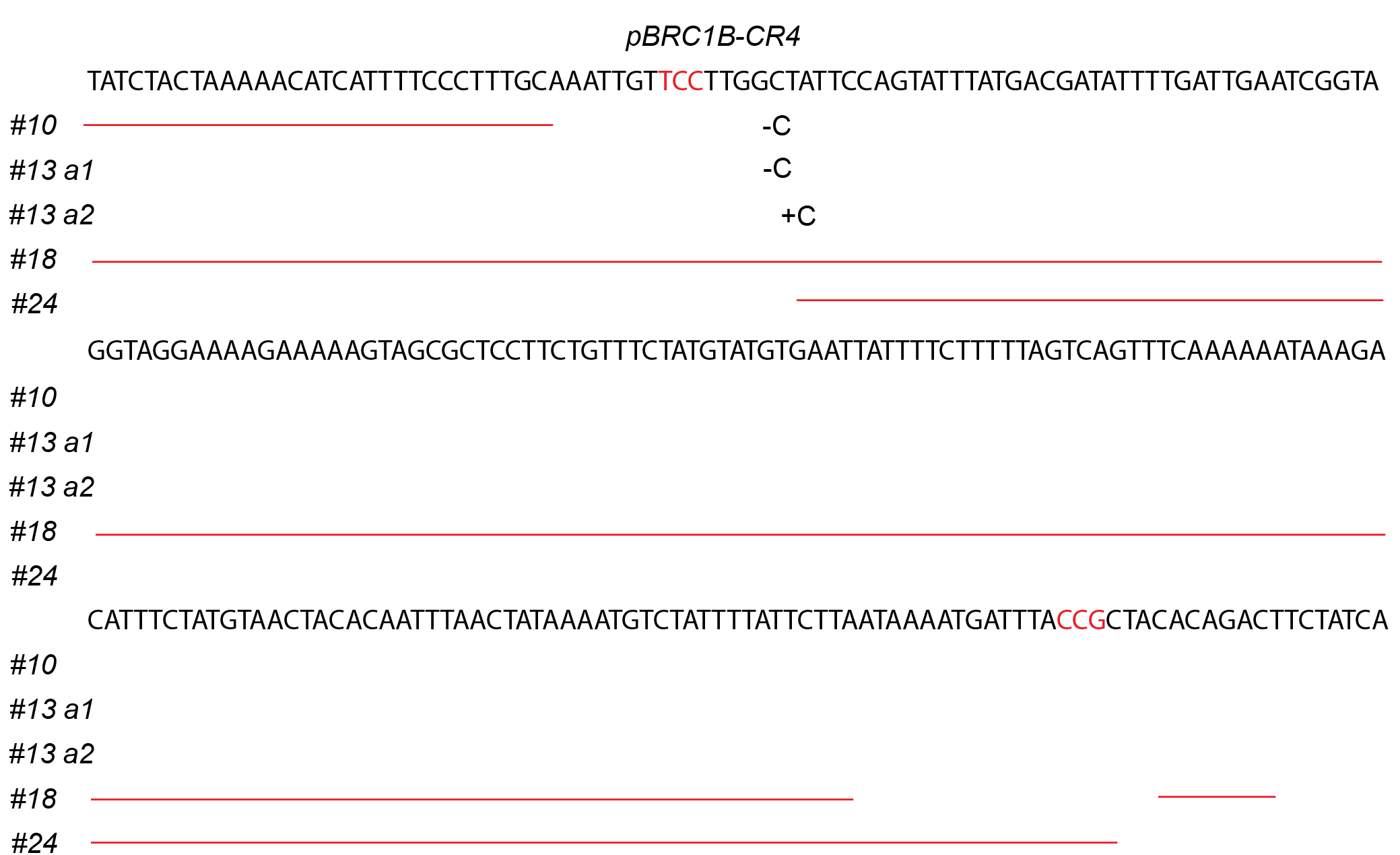
**

**
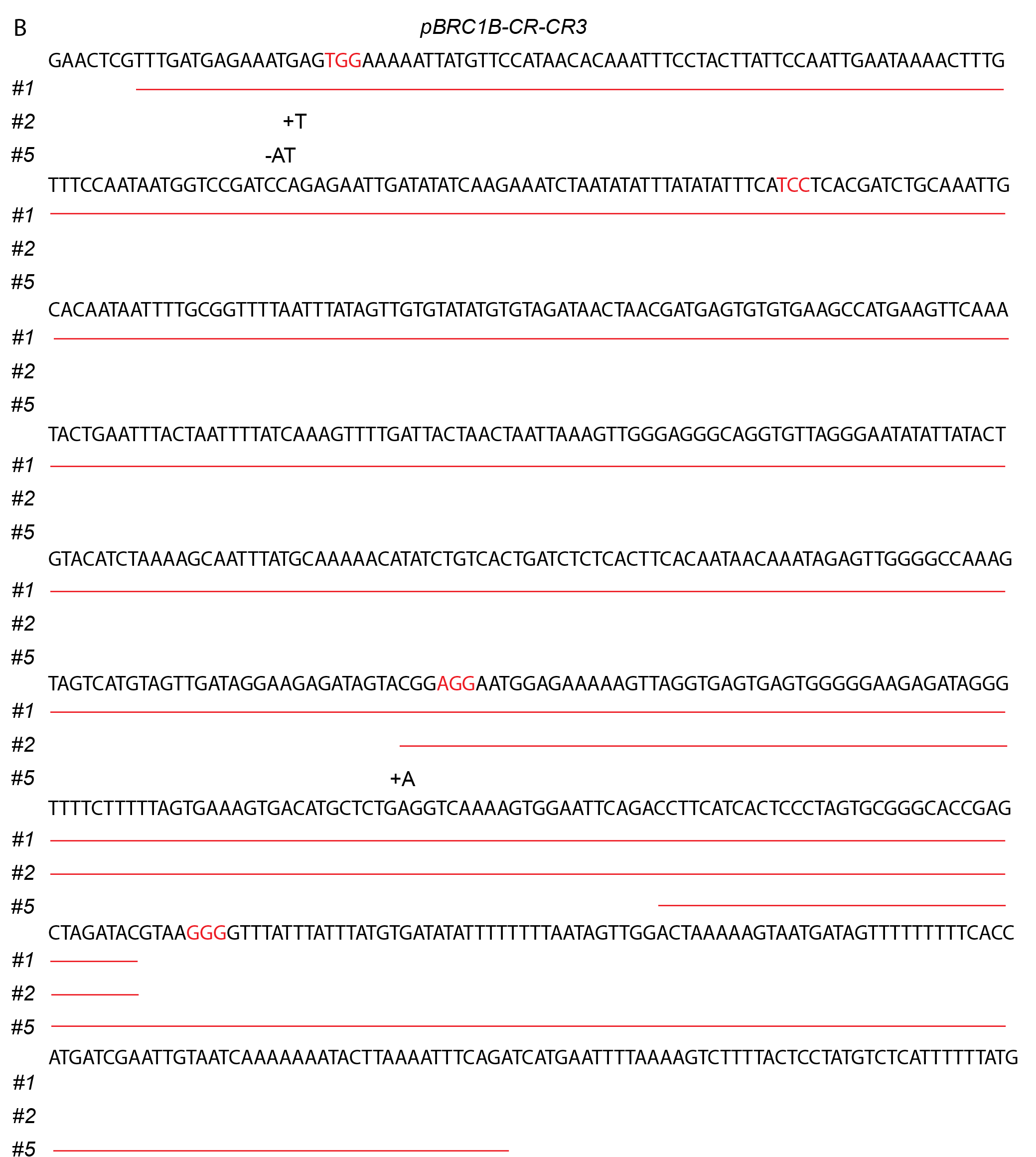
**

**
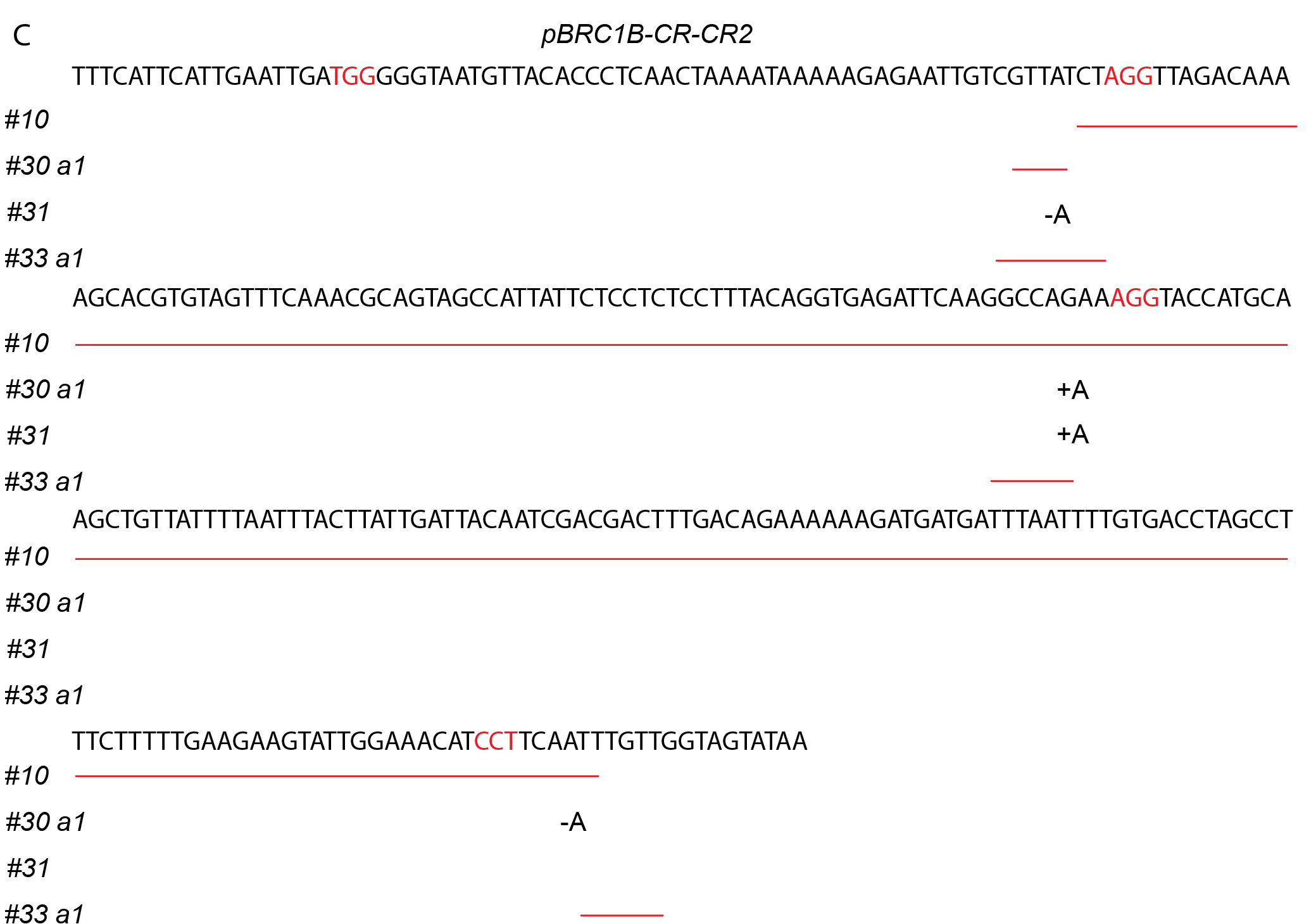
**

**
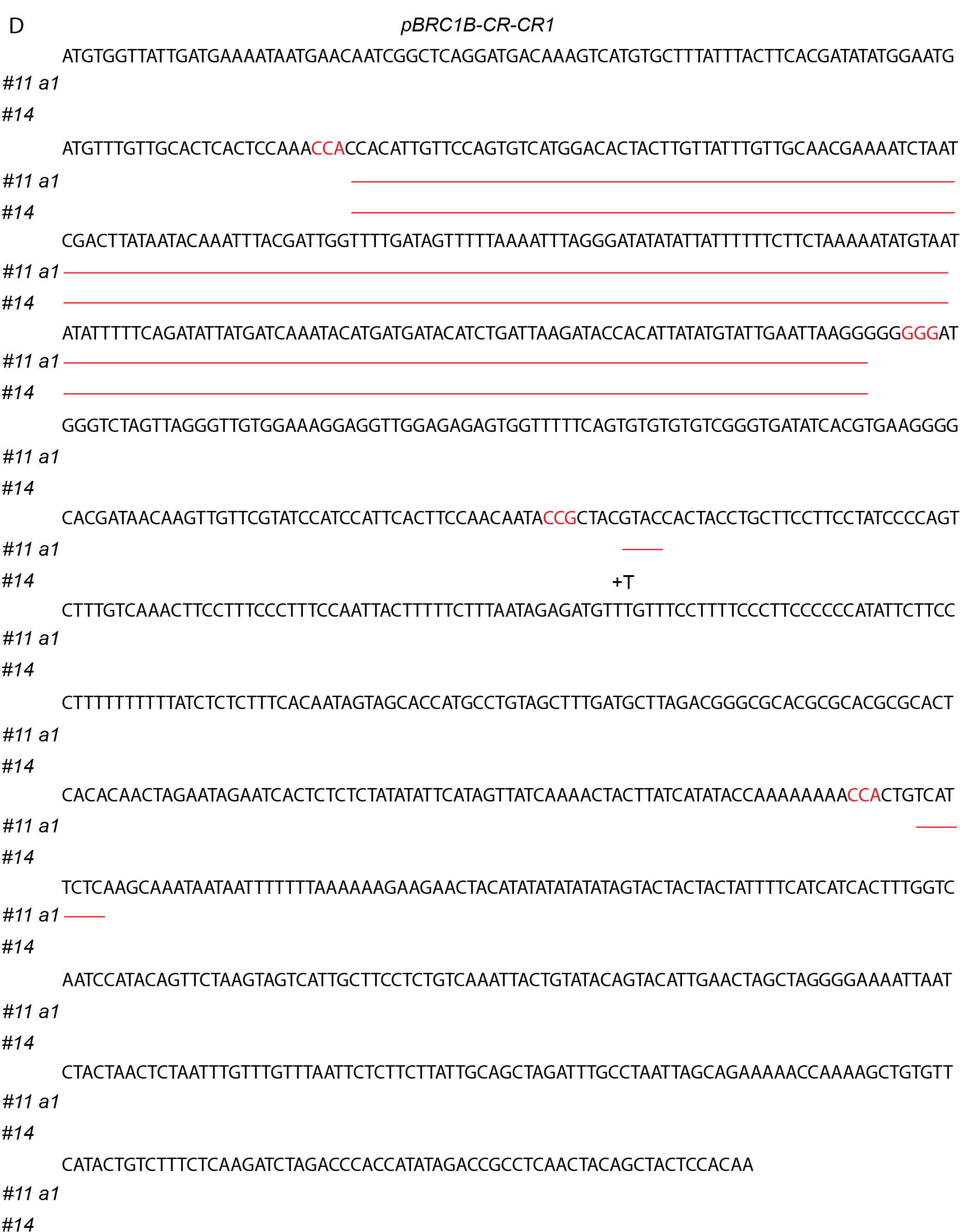
**

**Supplementary Figure S4** Full-length sequences of wild-type and *CR* mutant *SlBRC1B* promoter fragments. **(A-D)** Alignments between the respective wild-type *CR* and *CR4* (A), *CR3* (B), *CR2* (C) and *CR1* (D) mutants. PAM sites are highlighted in red and red lines under the sequences represent deletions in the mutants. Insertions are denoted by a plus (+) and deletions by a dash (-) followed by a number indicating their length.


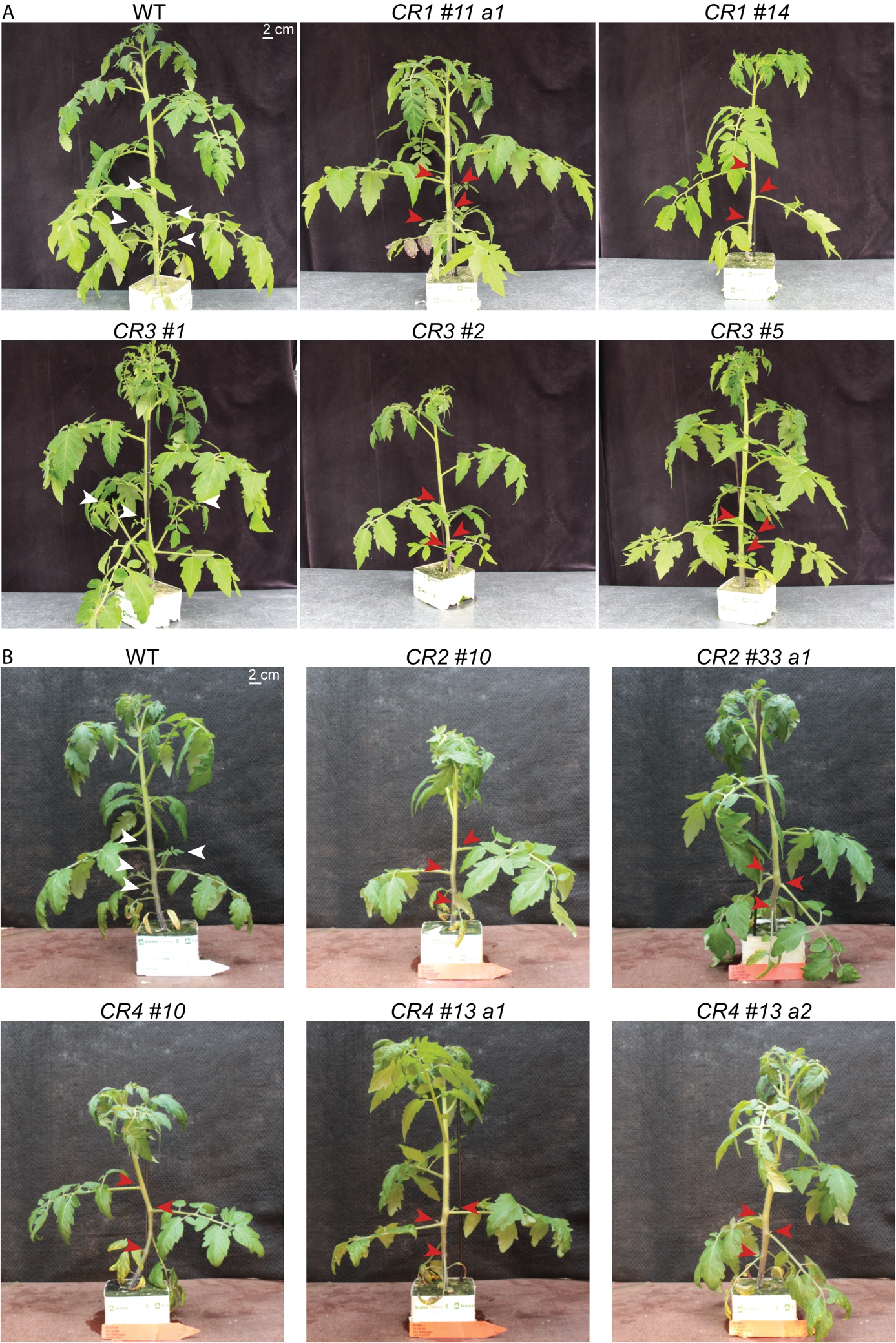


**Supplementary Figure S5** Representative phenotypes of *SlBRC1B* *CR* mutants at 6 WAS. **(A-B)** Bud outgrowth was analyzed in two independent experiments, one including *CR1* and *CR3* (A) and another including *CR2* and *CR4* (B), each with their respective wild-type controls. Basal axillary shoots are indicated by white arrows in the wild-type and *CR3 #1* mutant, showing a similar branching phenotype. Decreased bud outgrowth in the remaining *CR* mutants is indicated by red arrowheads. The scale bar in the wild-type image represents 2 cm and applies to all images within the corresponding experimental batch.

**Supplementary Table S1** Selected homologous sequences of SlBRC1B for phylogenetic footprinting. Homologs within the Solanaceae were identified using BLASTp searches. Homologs in other angiosperm species, collectively referred to as *TB1* clade, as indicated with an asterisk, were identified and selected based on information from publicly available functional studies. CYC: CYCLOIDEA, DIC: DICHOTOMA.

| Species name | Locus number | Gene ID | Clade |
| --- | --- | --- | --- |
| *Solanum lycopersicum* | Solyc06g069240 | *SlBRC1B* | *BRC1B*-subclade |
| *Solanum pennellii* | LOC107021853 | *SpCYC-like* |  |
| *Solanum tuberosum* | HM921051 | *StBRC1B* |  |
| *Capsicum annuum* | LOC107855370 | *CaCYC* |  |
| *Nicotiana sylvestris* | LOC104215100 | *NsCYC-like* |  |
| *Nicotiana tomentosiformis* | LOC104102210 | *NtCYC-like* |  |
| *Nicotiana attenuata* | OIT02861 | *NsTB1* |  |
| *Nicotiana attenuata* | LOC109224707 | *NaDIC-like* | *BRC1A*-subclade |
| *Solanum tuberosum* | HM921052 | *StBRC1A* |  |
| *Solanum pennellii* | LOC107013914 | *SpTCP12-like* |  |
| *Solanum lycopersicum* | Solyc03g119770 | *SlBRC1A* |  |
| *Arabidopsis thaliana* | AT3G18550 | *AtBRC1* | *TB1* clade* |
| *Arabidopsis thaliana* | AT1G68800 | *AtBRC2* |  |
| *Cucumis sativus* | Csa1G020890 | *CsBRC1* |  |
| *Zea mays* | Zm00001d033673 | *ZmTB1* |  |

**Supplementary Table S2** TOMTOM transcription factor (TF) consensus binding site analysis within identified MEME motifs M2-M10, located five kb upstream of *SlBRC1B*. TF consensus binding sites were searched for in MEME motifs m2-m10. m1 was not found five kb upstream of *SlBRC1B*. Clade information for each TF family was derived from a comprehensive literature search.

| MEME motif | TF family | Clade |
| --- | --- | --- |
| **m2** | B3-REM | Subgroup A and B |
|  | COL/BBX | Group V |
|  | DOF | Group A, B1, B2, C1, C2, D1, D2 |
|  | ERF | A-5 (Group II), B-1 (Group VIII), B-2 (Group VII), B-4 (Group X) |
|  | FAR1 | Subgroup IV |
|  | LBD | Class I |
|  | MYB | R2R3 |
|  | MYB-related | R-R-type |
|  | NAC (NTL) | Group II (ANAC001 clade) |
|  | Zinc finger C2H2 type | Subgroup A1 IDD domain clade B; subgroup C1-2i |
| **m3** | AP2/ERF | DREB |
|  | bHLH | Subclade 11 |
|  | E2F/DP | Group DEL |
|  | FAR1 | FAR1 subgroup IV |
|  | NAC | Group 1 (NAC2, ANAC011, ONAC003, NAM, OsNAC7), group 2 (ONAC003) |
|  | Zinc finger C2H2 type | Subgroups: C1-2i; AN1-like group I |
| **m4** | B3-REM | Subgroup A |
|  | COL/BBX | Group V |
|  | DOF | Group B1, D2 |
|  | SBP | SPL2 clade |
|  | Zinc finger C2H2 | Subgroup A1 IDD domain clade D |
| **m5** | B3-RAV | Subgroup NGA |
|  | bHLH | Subclade 11, 16 |
|  | bZIP | Group i |
|  | Zinc finger C2H2 type | Subgroup A1c; C1-2ic |
| **m6** | E2F/DP | Group E2F |
|  | Homeobox (HB) | HD-ZIP class I |
|  | TCP | Class I; class II CIN-clade |
| **m7** | bHLH | Subclade 11, 16 |
|  | CAMTA | Group B |
|  | G2-like | Group I |
|  | MYB | R2R3 |
|  | NAC | Group 1 (AtNAC3, NAP, ATAF) |
|  | WRKY | Group IIb |
|  | Zinc finger C2H2 type | Subgroup A1d WIP domain |
| **m8** | B3-LAV | Subgroup LEC2-ABI3 |
|  | MYB-related | CCA1-like |
|  | MADS | Type II MADS MIKC type clades: FLC/MAF and TT16 |
| **m9** | BES1/BZR1 | Cluster V, VI |
|  | bHLH | Subclade 18 |
|  | GRF | Group II (tomato article) |
|  | MADS | Type II MADS MIKC type clades: AGL6, AGL15 and TT16 |
|  |  | Type I MADS Malpha- and Mgamma-types |
|  | MYB-related | CCA1-like, R-R-type |
|  | NAC | Group 1 (OsNAC8, NAC2, NAM) |
|  | Trihelix | Subfamily beta |
| **m10** | B3-REM | Subgroup A and B |
|  | COL/BBX | Group V |
|  | DOF | Group A, B1, C2.1, D1, D2 |
|  | Homeobox (HB) | HD-ZIP class I subclass -beta, -sigma and -epsilon |
|  |  | HD-ZIP class II subclass alpha |
|  | ZF-HB | Clade II |
|  | MADS | Type II MADS MIKC type clades: AGL6, FLC/MAF and SVP |
|  | MYB | Clade I |
|  | MYB-related | R-R-type |
|  | NAC | Group II (ANAC001 clade) |
|  | Trihelix | Subfamily beta |
|  | WRKY | Group IIa/b/c |
|  | Zinc finger C2H2 type | Subgroup A1 IDD domain: clade A, B, C and D |

**Supplementary Table S3** Identification of TFs in Yeast one-hybrid screens between *CR4* and cDNA library of axillary buds. Gene IDs specify the characterized genes in tomato with corresponding references. For genes that are not previously studied in tomato, such as *CZFP1* and *MYB1R1* (indicated by an asterisk (*)) the ITAG4.0 annotation is used. Respective studies identified phylogenetic relationships between tomato and Arabidopsis, enabling the classification of Arabidopsis homologs. Based on phylogenetic analysis (Bhattacharjee et al., 2017; Hu et al., 2019; Khatun et al., 2017; Wang et al., 2019; P. Zhao et al., 2014), identified TFs were further categorized into TF families and specific subgroups in this study. MEME motifs illustrate the presence of consensus binding sites for individual TFs. GRF: GROWTH REGULATING FACTOR, CZFP: C2H2-type zinc finger protein, ZAT: ZINC FINGER PROTEIN, NACMTF: NAC MEMBRANE-BOUND TF, NTL: NAC WITH TRANSMEMBRANE MOTIF 1-LIKE, SEP: SEPALLATA, MB1R1: MYB-related TF, MYBD: MYB-LIKE DOMAIN.

| Sol ID | Gene ID | Arabidopsis homologs | TF family | Clade information | MEME motif sites |
| --- | --- | --- | --- | --- | --- |
| Solyc01g091540 | SlGRF10 (Khatun et al., 2017) | AtGRF9 | GRF | Group II | m9 |
| Solyc04g077980 | SlCZFP1*  (Hu et al., 2019) | AtZAT10 | C2H2 | Class II | m2, m3, m6, m10 |
| Solyc03g080090 | SlNACMTF2  (Bhattacharjee et al., 2017) | AtNTL6 | NAC | Group I, TIP clade | m2, m3, m7, m9, m10 |
| Solyc05g015750 | SlSEP3  (Wang et al., 2019) | AtSEP3 | MADS type II | SEP | m8, m9 |
| Solyc03g113620 | SlMYB1R1* | AtMYBD | MYB-related | MYB-related CCA1-like | m2, m8, m9 |

**Supplementary Table S4** List of primers used in this study. Genotyping primers were used for Sanger sequencing, except for *CR1*, marked with an asterisk (*).

| **Experiment** | **Target** | **Forward (5'-3')** | **Reverse (3'-5')** |
| --- | --- | --- | --- |
| CRISPR mutagenesis | *CR1* sgRNA1 | ATTGATGACACTGGAACAATGTGG | AAACCCACATTGTTCCAGTGTCAT |
|  | *CR1* sgRNA2 | ATTGTATGTATTGAATTAAGGGGG | AAACCCCCCTTAATTCAATACATA |
|  | *CR1* sgRNA3 | ATTGAAGCAGGTAGTGGTACGTAG | AAACCTACGTACCACTACCTGCTT |
|  | *CR1* sgRNA4 | ATTGTATTTGCTTGAGAATGACAG | AAACCTGTCATTCTCAAGCAAATA |
|  | *CR2* sgRNA1 | ATTGGGTTTCATTCATTGAATTGA | AAACTCAATTCAATGAATGAAACC |
|  | *CR2* sgRNA2 | ATTGAAAGAGAATTGTCGTTATCT | AAACAGATAACGACAATTCTCTTT |
|  | *CR2* sgRNA3 | ATTGGGTGAGATTCAAGGCCAGAA | AAACTTCTGGCCTTGAATCTCACC |
|  | *CR2* sgRNA4 | ATTGTTATACTACCAACAAATTGA | AAACTCAATTTGTTGGTAGTATAA |
|  | *CR3* sgRNA1 | ATTGCTCGTTTGATGAGAAATGAG | AAACCTCATTTCTCATCAAACGAG |
|  | *CR3* sgRNA2 | ATTGTGTGCAATTTGCAGATCGTG | AAACCACGATCTGCAAATTGCACA |
|  | *CR3* sgRNA3 | ATTGGATAGGAAGAGATAGTACGG | AAACCCGTACTATCTCTTCCTATC |
|  | *CR3* sgRNA4 | ATTGGCACCGAGCTAGATACGTAA | AAACTTACGTATCTAGCTCGGTGC |
|  | *CR4* sgRNA1 | ATTGCATAGGCTCTCATTGTCACC | AAACGGTGACAATGAGAGCCTATG |
|  | *CR4* sgRNA2 | ATTGGCCCAAATTAGGGCACACCA | AAACTGGTGTGCCCTAATTTGGGC |
|  | *CR4* sgRNA3 | ATTGCATAAATACTGGAATAGCCA | AAACTGGCTATTCCAGTATTTATG |
|  | *CR4* sgRNA4 | ATTGAATGATAGAAGTCTGTGTAG | AAACCTACACAGACTTCTATCATT |
| CRISPR genotyping | *CR1** | TACAATCGACGACTTTGACAG | AGCTGTAGTTGAGGCGGTC |
|  | *CR2* | AAAGCGAGCTTCTGTTCACG | TGTGCTCGTATAGAACTTGGG |
|  | *CR3* | ACCGCTACACAGACTTCTATC | AGCGTCGATTGATACTCTTTG |
|  | *CR4* | GAGTGACTTACTGACACCATTTC | CCACTCATTTCTCATCAAACGAG |
| CRISPR amplicon sequencing | *CR1** | CGATGTAAGGATAGAATATG | GTGGAGTAGCTGTAGTTGAGG |
| Y1H cloning | *CR4* | GGGGACAAGTTTGTACAAAAAAGCAGGCTCCTTTACATTATTAATGTTTGC | GGGGACCACTTTGTACAAGAAAGCTGGGTTCTGTGTAGCGGTAAATC |
| Y1H genotyping and sequencing identified clones | *CR4* | GCCGACAACCTTGATTGGAGAC | CGGTCCGAACCTCATAACAACTC |
| RT-qPCR | *BRC1B* | TGGTGCAATTTGTGCATCTA | ATCTTGAGCGGTTTCCTTGT |
|  | *CAC* | CCTCCGTTGTGATGTAACTGG | ATTGGTGGAAAGTAACATCATCG |
|  | *EXPRESSED* | CCCGAGGAAGCTGGTCTAC | CAAACAGGCCATGCAACACT |

# References

Bhattacharjee, P., Das, R., Mandal, A., & Kundu, P. (2017). Functional characterization of tomato membrane-bound NAC transcription factors. *Plant Molecular Biology*, *93*(4–5), 511–532. https://doi.org/10.1007/s11103-016-0579-z

Hu, X., Zhu, L., Zhang, Y., Xu, L., Li, N., Zhang, X., & Pan, Y. (2019). Genome-wide identification of C2H2 zinc-finger genes and their expression patterns under heat stress in tomato (Solanum lycopersicum L.). *PeerJ*, *2019*(11). https://doi.org/10.7717/peerj.7929

Khatun, K., Robin, A. H. K., Park, J. I., Nath, U. K., Kim, C. K., Lim, K. B., Nou, I. S., & Chung, M. Y. (2017). Molecular characterization and expression profiling of tomato GRF transcription factor family genes in response to abiotic stresses and phytohormones. *International Journal of Molecular Sciences*, *18*(5). https://doi.org/10.3390/ijms18051056

Wang, Y., Zhang, J., Hu, Z., Guo, X., Tian, S., & Chen, G. (2019). Genome-wide analysis of the MADS-box transcription factor family in Solanum lycopersicum. *International Journal of Molecular Sciences*, *20*(12). https://doi.org/10.3390/ijms20122961
